# Rb-Mediated Neuronal Differentiation through Cell-Cycle–Independent Regulation of E2f3a

**DOI:** 10.1371/journal.pbio.0050179

**Published:** 2007-07-03

**Authors:** Danian Chen, Rene Opavsky, Marek Pacal, Naoyuki Tanimoto, Pamela Wenzel, Mathias W Seeliger, Gustavo Leone, Rod Bremner

**Affiliations:** 1 Genetics and Development Division, Toronto Western Research Institute, University Health Network, University of Toronto, Ontario, Canada; 2 Department of Ophthalmology and Visual Science, University of Toronto, Ontario, Canada; 3 Department of Laboratory Medicine and Pathobiology, University of Toronto, Ontario, Canada; 4 Human Cancer Genetics Program, Department of Molecular Virology, Immunology and Medical Genetics, Ohio State University, Columbus, Ohio, United States of America; 5 Department of Molecular Genetics, Ohio State University, Columbus, Ohio, United States of America; 6 Comprehensive Cancer Center, Ohio State University, Columbus, Ohio, United States of America; 7 Ocular Neurodegeneration Research Group, Centre for Ophthalmology, Institute for Ophthalmic Research, University of Tuebingen, Germany; Cambridge University, United Kingdom

## Abstract

It has long been known that loss of the retinoblastoma protein (Rb) perturbs neural differentiation, but the underlying mechanism has never been solved. Rb absence impairs cell cycle exit and triggers death of some neurons, so differentiation defects may well be indirect. Indeed, we show that abnormalities in both differentiation and light-evoked electrophysiological responses in Rb-deficient retinal cells are rescued when ectopic division and apoptosis are blocked specifically by deleting E2f transcription factor (E2f) 1. However, comprehensive cell-type analysis of the rescued double-null retina exposed cell-cycle–independent differentiation defects specifically in starburst amacrine cells (SACs), cholinergic interneurons critical in direction selectivity and developmentally important rhythmic bursts. Typically, Rb is thought to block division by repressing E2fs, but to promote differentiation by potentiating tissue-specific factors. Remarkably, however, Rb promotes SAC differentiation by inhibiting E2f3 activity. Two E2f3 isoforms exist, and we find both in the developing retina, although intriguingly they show distinct subcellular distribution. E2f3b is thought to mediate Rb function in quiescent cells. However, in what is to our knowledge the first work to dissect E2f isoform function in vivo we show that Rb promotes SAC differentiation through E2f3a. These data reveal a mechanism through which Rb regulates neural differentiation directly, and, unexpectedly, it involves inhibition of E2f3a, not potentiation of tissue-specific factors.

## Author Summary

The retinoblastoma protein (Rb), an important tumor suppressor, blocks division and death by inhibiting the E2f transcription factor family. In contrast, Rb is thought to promote differentiation by potentiating tissue-specific transcription factors, although differentiation defects in Rb null cells could be an indirect consequence of E2f-driven division and death. Here, we resolve different mechanisms by which Rb controls division, death, and differentiation in the retina. Removing E2f1 rescues aberrant division of differentiating Rb-deficient retinal neurons, as well as death in cells prone to apoptosis, and restores both normal differentiation and function of major cell types, such as photoreceptors. However, Rb-deficient starburst amacrine neurons differentiate abnormally even when E2f1 is removed, providing an unequivocal example of a direct role for Rb in neuronal differentiation. Rather than potentiating a cell-specific factor, Rb promotes starburst cell differentiation by inhibiting another E2f, E2f3a. This cell-cycle–independent activity broadens the importance of the Rb–E2f pathway, and suggests we should reassess its role in the differentiation of other cell types.

## Abstract 3

The retinoblastoma protein (Rb), a tumor suppressor, promotes the differentiation of starburst amacrine cells in the retina by inhibiting the transcription factor E2f3a, whereas it suppresses retinal cell division and death by inhibiting E2f1.

## Introduction

**Figure 1:**
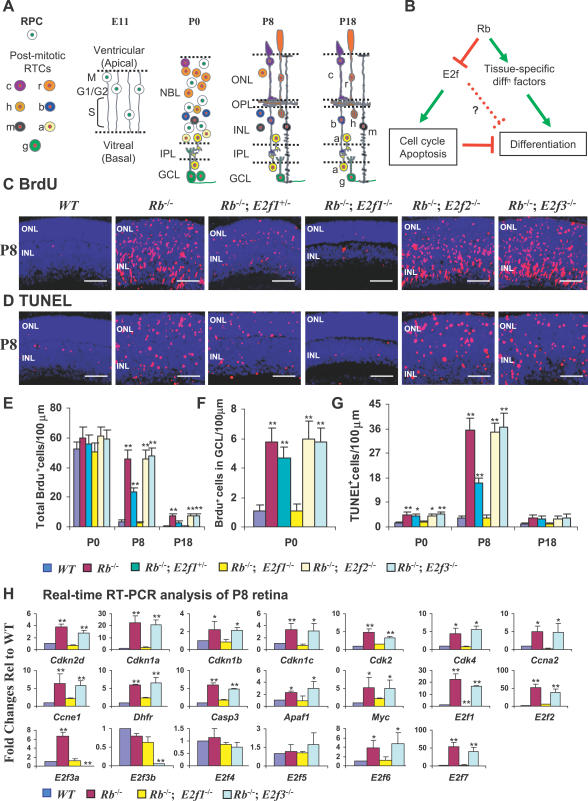
*E2f1,* but Not *E2f2* or *E2f3,* Loss Rescues Ectopic Division and Cell Death in the *Rb* KO Retina (A) Retinal development. At E11 the retina is a NBL of dividing RPCs (white circle, green nuclei). RPC cell bodies oscillate along processes as they progress through the cell cycle. By P0 the NBL contains both RPCs and post-mitotic RTCs (coloured circles, red nuclei) and is separated from the GCL by the IPL. By P8 there are no RPCs, fewer RTCs, an OPL, and more differentiated rods (r) and cones (c) in the ONL; horizontal (h), bipolar (b), Müller (m), and amacrine (a) cells in the INL; and ganglion (g) and displaced amacrine cells in the GCL. Development is complete by ~P18. (B) Rb is thought to regulate cell cycle and apoptosis by repressing E2fs, but to promote differentiation by potentiating tissue-specific transcription factors. However, Rb loss could also perturb differentiation through the indirect effects of abnormal division or death, and/or through direct regulation of differentiation genes by E2fs. (C and D) Horizontal retinal sections of the indicated genotypes and ages were stained for nuclei (DAPI, blue), and (C) S-phase (anti-BrdU, red) or (D) apoptosis (TUNEL, red). Scale bars are 50 μm. (E–G) Quantification of (E) all BrdU^+^ cells, (F) ectopic BrdU^+^ cells in GCL at P0, and (G) total TUNEL^+^ cells. (H) Real-time RT-PCR analysis of E2fs and E2f target genes in P8 retinas of the indicated genotypes. Error bars represent SD of measurements from three animals, and asterisks indicate a significant difference between the WT and indicated genotypes (*, p <0.05; **; p <0.01; ANOVA and Tukey HSD test for [E–G] and Fisher test for [H]).

The simplicity of the retina makes it an ideal tissue to study neurogenesis. Its development proceeds through three overlapping steps starting with retinal progenitor cell (RPC) proliferation, followed by birth of post-mitotic retinal transition cells (RTCs, also referred to as precursors), and ending with terminal differentiation of seven major cell types (Figure 1A) [1]. RPCs are multipotent and exit the cell cycle to generate different RTCs at specific time periods in development [1]. This process of RTC “birth” requires coupling of differentiation and cell cycle exit. Once born, post-mitotic RTCs migrate and form different retinal layers. Rods and cones make up the outer nuclear layer (ONL); horizontal, bipolar, and amacrine cells, as well as Müller glia cell bodies, reside in the inner nuclear layer (INL); and ganglion and displaced amacrine cells form the ganglion cell layer (GCL) (Figure 1A). The outer plexiform layer (OPL) and inner plexiform layer (IPL) house synaptic connections separating the ONL/INL and INL/GCL, respectively.

The retinoblastoma protein (Rb) is critical for cell cycle exit during retinal transition cell birth. *Rb* knockout (KO) RTCs continue to proliferate inappropriately and some (rod, ganglion, and bipolar cells) die by apoptosis [2,3]. Rb controls the cell cycle by binding and inhibiting E2f transcription factors (E2fs) (Figure 1B), first defined as transcription factors that bind adenoviral E2 regulatory elements and subsequently shown to be critical cell cycle regulators [4,5]. E2fs bind to DNA as heterodimers with proteins of the related Tfdp family. E2f1, E2f2, and E2f3a are “activating E2fs” that are required for fibroblast division. They are strong transcriptional activators that can drive G0 fibroblasts into cycle, and are inhibited when bound to Rb [4,5]. Ectopic division in *Rb* KO embryos can be rescued to various extents in different tissues by knocking out *E2f1, E2f2,* or *E2f3* [6–9], but which member(s) drive division in *Rb* KO RTCs is unknown. Other members of the family, such as E2f4 and E2f5, are known as “repressive E2fs” because they are weak activators and appear to be primarily involved in gene silencing in quiescent or differentiated cells.

Activating E2fs may also promote apoptosis in the *Rb* KO retina (Figure 1B). Originally, E2f1 was considered the primary pro-apoptotic member of the family [10]. However, this view was reevaluated when it was shown that either *E2f1* or *E2f3* deletion rescues apoptosis in the developing central nervous system (CNS) of *Rb* KO embryos [6,11]. Subsequently, CNS apoptosis was shown to be an indirect result of placental defects and probable hypoxia [12–14]. Indeed, E2f3-induced apoptosis in fibroblasts has recently been shown to require E2f1 [15]. Thus, it is controversial whether E2f3 is required for apoptosis of any *Rb* KO cell type. Determining which activating E2fs promote death in distinct *Rb* KO tissues requires conditional rather than germ line models of *Rb* deletion to avoid secondary indirect effects (such as hypoxia).

E2f family diversity is expanded by E2f3 isoforms. Alternative promoters generate two forms (a and b) that are identical except for distinct first exons [16]. E2f3a is a strong activator, and, like other activating E2fs, its expression is induced when quiescent cells are stimulated to divide [16]. E2f3b, like repressive E2fs, is present in both quiescent and dividing cells, and in quiescent fibroblasts it associates primarily with Rb, suggesting that it mediates repression [16–18]. Indeed, silencing the Cdkn2d (p19^Arf^) locus in unstressed cells relies on E2f3b [19]. Other E2fs may also exist in isoforms since at least two mRNA species have been detected for E2f1 and E2f2 [16]. The roles of E2f isoforms in vivo are unknown.

E2fs are also regulated by subcellular localization. Although this feature has been best characterized for repressive E2fs [20–22], it also affects activating E2fs [23–25]. The distribution of E2f isoforms has never been assessed.

It has been known for many years that Rb loss perturbs neuronal differentiation [26–29]. However, prior work could not exclude the possibility that differentiation defects are simply an indirect consequence of abnormal division and death. If Rb does regulate differentiation directly it is unclear whether it does so in all or a subset of neurons. Moreover, the mechanism has never been solved. In other cell types where Rb may promote differentiation directly, such as muscle and bone, it seems to do so through E2f-independent means by potentiating tissue-specific transcription factors (Figure 1B) [30–33]. In the retina, others have noted abnormally shaped *Rb* KO rods and have suggested Rb may directly promote their morphogenesis by activating retina-specific factors [29]. However, differentiation defects in any *Rb* KO neuron could be an indirect effect of ectopic division and/or apoptosis (Figure 1B). Thus, it is critical to study differentiation of *Rb* KO cells in the absence of ectopic proliferation and death.

Here, we establish that Rb suppresses RTC division and death by inhibiting E2f1, not E2f2 or E2f3. When these defects were rescued, most retinal neurons, including rods, survived, differentiated, and functioned normally. Thus, unexpectedly, retina-specific differentiation factors function independently of Rb. However, comprehensive assessment of the *Rb*/*E2f1* double-null rescued retina revealed a differentiation defect in cholinergic starburst amacrine cells (SACs). Recent breakthroughs have revealed that these interneurons are critical for direction selectivity and developmentally important rhythmic bursts [34–36]. However, their differentiation is poorly understood. Contrary to the prevailing view that Rb promotes differentiation through E2f-independent tissue-specific transcription factors, we show that Rb facilitates SAC development through E2f3. Defects in *Rb* null SACs correlated with specific E2f3 expression in these cells, and E2f3 expression was absent in neurons that differentiated without Rb. E2f3 is also present in a specific subset of other CNS neurons, implying that this may be a general mechanism by which Rb facilitates neurogenesis. To define the mechanism in even more detail, we determined which E2f3 isoform Rb targets to control SAC differentiation. E2f3b mediates Rb function in quiescent fibroblasts [19], yet no prior studies to our knowledge have dissected E2f3a or E2f3b functions in vivo. Using an isoform-specific null mouse we show that Rb drives SAC differentiation through E2f3a. Thus, independent of E2f1-mediated effects on division and death, Rb does regulate neuronal differentiation, but only in specific neurons and, unexpectedly, through E2f3a, not tissue-specific differentiation factors.

## Results

### Rb Regulates Division and Death through E2f1

We used the *α-Cre* transgene to delete floxed *Rb* exon 19 at embryonic day (E) 10 in peripheral retina [2]. *Rb^loxP/loxP^;α-Cre* mice were bred with strains lacking *E2f1* or *E2f2* in the germ line, or a strain carrying a floxed *E2f3* allele [5]. *Rb^loxP/loxP^;E2f1^+/−^* and *Rb^loxP/loxP^;E2f1^+/−^;α-Cre* mice were bred to produce *Rb^loxP/loxP^;E2f1^−/−^;α-Cre* mice at a frequency of 1/8 and littermate controls at the same or higher (1/4) frequency. For simplicity we will refer to the *Rb^loxP/loxP^;E2f1^−/−^;α-Cre* peripheral retina as the *Rb/E2f1* double knockout (DKO) retina. Similar strategies were employed in the case of *E2f2* or *E2f3*. Cre-mediated excision of *Rb* and *E2f3* alleles in the retina was confirmed by PCR as described previously [2,5].

To measure ectopic cell division, mice were pulse-labelled with bromodeoxyuridine (BrdU) 2 h before sacrifice and the peripheral retina analyzed for BrdU incorporation by immunofluorescence. As reported before [2,3], *Rb* KO retinas exhibited both spatial and temporal ectopic DNA synthesis (Figures 1C and S1A). This is easily detected at E14, E16, and postnatal day (P) 0 in the inner retina where abnormal BrdU^+^ ganglion and amacrine RTCs are located, or on the outermost region of the P0 retina, where BrdU^+^ photoreceptor RTCs reside (Figures S1A and S2, arrows) [2]. Ectopic RTC division in *Rb* KO retinas is even more obvious at P8 or P18, when division is completed in wild-type (WT) retina (Figures 1C and S1A). Strikingly, the ectopically positioned S-phase cells at E14, E16, and P0 and all the abnormal division at P8 and P18 were completely suppressed in the *Rb/E2f1* DKO retina (Figures 1C, 1E, 1F, S1A, and S2). In contrast, deletion of *E2f2* or *E2f3* had no effect at any stage of development. Analysis of mitotic cells with anti–phosphohistone 3 (PH3)–specific antibodies confirmed that loss of *E2f1,* but not *E2f2* or *E2f3,* suppressed ectopic division (Figure S3). Deleting one *E2f1* allele partially suppressed ectopic S-phase and mitosis in *Rb* KO RTCs (Figures 1C, 1E, 1F, S1A, S2, and S3), suggesting that E2f1 drives ectopic division in *Rb* KO RTCs in a dose-dependent fashion. These data contrast with previous findings in the lens and CNS of *Rb* KO embryos, where deletion of any activating E2f suppresses ectopic division to some extent [6–9].

**Figure 2:**
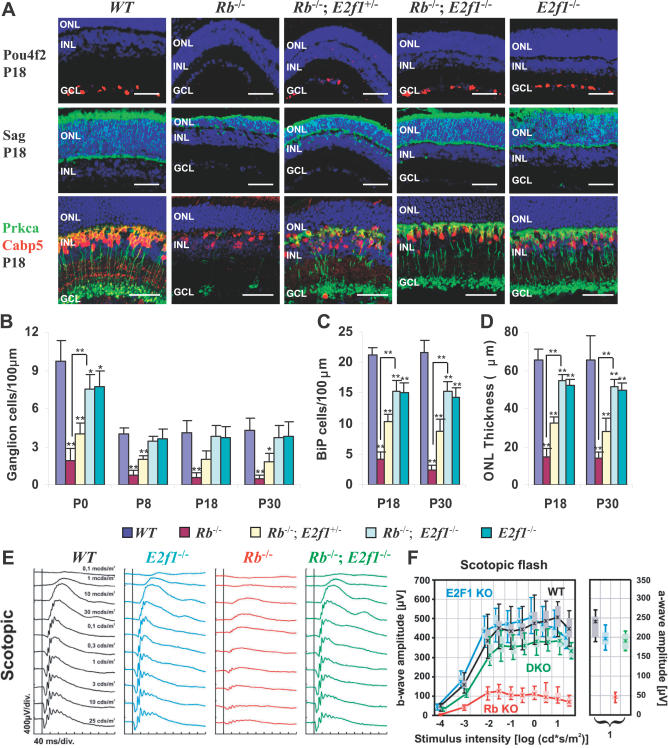
*E2f1* Deletion Rescues Ganglion, Rod, and Bipolar Cells in the *Rb* KO Retina (A) Horizontal retinal sections from mice of the indicated ages and genotypes were stained for nuclei (DAPI, blue) and markers that detect ganglion cells (Pou4f2, red), rods and cones (Sag [rod arrestin], green), and rod bipolar cells (Prkca, green, and Cabp5, red). Scale bars are 50 μm. (B) Quantification of Pou4f2^+^ ganglion cells. (C) Quantification of Prkca^+^ and Cabp5^+^ bipolar cells. (D) Thickness of the ONL, which represents the number of rods. Error bars represent SD of measurements from three animals, and asterisks indicate a significant difference between retinas of WT and the indicated genotypes, unless indicated otherwise by connecting lines (*, p < 0.05; **; p < 0.01; ANOVA and Tukey HSD test). (E and F) ERGs were recorded from the indicated genotypes under dark-adapted (scotopic) conditions, and (E) intensity series and (F) b-wave amplitudes as a function of the logarithm of the flash intensity were determined. (F) Further illustrates that the relative influence of the mutations on the photoreceptors (indicated by the saturated a-wave amplitude, right graph) was not substantially different from their effect on the b-wave response (dominated by the bipolars, left graph) at the same intensity of 10 cd·s/m^2^.

Loss of *Rb* in the retina results in considerable RTC apoptosis, eliminating most bipolar and ganglion cells as well as many rods (Figure 2A–2D) [2,3]. The loss of *Rb* KO rods is evident from the thinner ONL, and the death of these cells as well as bipolar and ganglion neurons can be detected directly by double labelling for apoptotic and cell-type-specific markers [2] (M. P. and R. B., unpublished data). Loss of peripheral *Rb* KO ganglion cells is also evident from thinning of the optic nerve (D. C. and R. B., unpublished data). Deleting *E2f1,* but not *E2f2* or *E2f3,* blocked this ectopic cell death in a dose-dependent fashion (Figures 1D, 1G, and S1B).

To investigate the molecular mechanism that underlies the unique role of E2f1, we assessed the expression of known E2f targets as well as other genes that regulate the cell cycle and apoptosis. Numerous positive and negative cell cycle and apoptotic regulators were up-regulated in the *Rb* KO retina (Figure 1H). Among the E2f family, *E2f1, E2f2, E2f3a,* and *E2f7* were induced following Rb loss, but *E2f3b, E2f4,* and *E2f5* were unaffected. Consistent with the BrdU and terminal dUTP nick-end labelling (TUNEL) analyses, *E2f1* deletion specifically reversed all these molecular defects, but *E2f3* deletion had no effect (Figure 1H).

### Normal Differentiation in the *Rb/E2f1* DKO Retina

Because *E2f1* deletion blocks abnormal division and death in the *Rb* KO retina, the *Rb/E2f1* DKO retina provided a unique opportunity to evaluate whether Rb controls differentiation independent of cell cycle effects. The *Rb/E2f1* DKO retina had many Sag^+^ (S-antigen/rod arrestin) photoreceptors, Pou4f2^+^ (Brn3b) ganglion cells, and numerous Prkca^+^/Cabp5^+^ bipolar neurons (Figure 2A–2D). In contrast, there was no such rescue of cell types in *Rb/E2f2* or *Rb/E2f3* DKO retinas (Figure S4). Analysis with general neuronal markers Mtap2 (MAP2) and Snap25, as well as other markers expressed in bipolar cells (Chx10, Rcvrn, Vsx1, Tacr3, and Atp2b1) and rod photoreceptors (Rho and Rcvrn) confirmed rescue of the *Rb/E2f1* DKO retina (Table S1). Moreover, neurons exhibited the same complex morphology as in WT retina. Bipolar cell bodies were located in the INL and had ascending and descending processes ending in the OPL and IPL, respectively (Figure 2A). In addition, the *Rb/E2f1* DKO retina had a healthy appearing ONL consisting of morphologically normal rods with descending processes ending in the OPL and ascending processes that terminated in inner and outer segments (Figure 2A). It was suggested that Rb might regulate photoreceptor differentiation, possibly through rod-specific transcription factors (Figure 1B) [29]. However, if Rb does regulate photoreceptor differentiation, it does so by inhibiting E2f1, not by potentiating rod differentiation factors, such as Otx2, Crx, or Nrl. It is impossible to tell whether E2f1 perturbs differentiation directly, by affecting the expression of genes that modulate maturation, and/or indirectly through its effects on proliferation and survival (Figure 1B).

As with ectopic division and apoptosis (Figure 1C and 1D), the rescue of *Rb* KO retinal bipolar, ganglion, and rod cells was dependent on *E2f1* dose (Figure 2A–2D). Separate from its role in driving ectopic division of *Rb* KO RTCs, E2f1 also promotes normal RPC division since in its absence RPC proliferation drops ~2-fold (D. C. and R. B., unpublished data). This modest reduction of RPC numbers in the absence of *E2f1* accounts for the slight reduction in the number of ganglion cells at P0, in the number of bipolar cells at P18 or P30, and in the thickness of the ONL at P18 or P30 in the *E2f1* KO and *Rb/E2f1* DKO retina (Figure 2B–2D). The morphology of *E2f1* KO neurons was WT (Figure 2A). Despite a slight drop in absolute cell numbers, the proportion of *Rb/E2f1* DKO and *E2f1* KO bipolar cells was the same as WT (data not shown). Slightly reduced cell numbers were not due to residual RTC death since we have not observed ectopic apoptosis at any embryonic or postnatal stage in the developing *Rb/E2f1* DKO retina (Figures 1D, 1G, and S2). Moreover, deleting *Ccnd1,* which acts upstream of Rb proteins, also reduces RPC number, but does not suppress any defect in the *Rb* KO retina (D. C. and R. B., unpublished data). Thus, slightly reduced RPC division and dramatic rescue of severe defects in *Rb* KO RTCs are distinct effects stemming from the deletion of *E2f1*.

### Normal Function of the *Rb/E2f1* DKO Retina

The discovery that *E2f1* loss rescues even the morphology of *Rb* KO neurons is surprising because Rb is thought to regulate differentiation primarily through E2f-independent pathways [30–33]. However, normal morphology may not equate to completely normal differentiation. Thus, we compared the electroretinograms (ERGs) of adult WT *(α-Cre), E2f1^−/−^, α-Cre;Rb^loxP/loxP^,* and *α-Cre;Rb^loxP/loxP^;E2f1^−/−^* mice. ERGs functionally assess visual signalling in the mammalian retina from photoreceptors to amacrine cells (but usually not gangion cells), and are dominated by rod and cone bipolar cells. Typically, an ERG signal begins with a negative deflection initiated by the photoreceptors (the a-wave), which is terminated by a large positive deflection due to the activation of ON bipolar cells (the b-wave).

Responses to dim light in dark-adapted (scotopic) conditions specifically assess the rod system, and were defective in the *Rb* KO retina (Figure 2E). The substantial reduction of both a- and b-waves is consistent with rod and bipolar cell apoptosis [2]. The sensitivity of the residual response appeared unchanged, suggesting it arose from the Cre-negative portions of the retina. Responses were about the same in the WT and *E2f1* KO retina, and, most importantly, also the *Rb/E2f1* DKO response median lay at the lower end of the normal range for most intensities (Figure 2F). Thus, *E2f1* deletion almost completely rescued the rod system in the *Rb* KO retina.

Light-adapted (photopic) recordings to specifically assess the cone system yielded comparable results. Cones represent only 3% of photoreceptors and, unlike rods, develop without *Rb,* but they require rods for survival, and in the *Rb* KO retina, they have abnormal morphology and their synaptic targets, bipolar cells, are much depleted [2]. The photopic response, a product of cone and mainly bipolar activity, was much reduced by *Rb* loss, but was rescued considerably in the *Rb/E2f1* DKO retina (Figure S5). Again, the median amplitude lay at the lower end of the *E2f1* KO range. The photopic response in *E2f1* KO mice was slightly reduced relative to WT (Figure S5B), possibly because E2f1 is required for maximal expansion of embryonic RPCs, and the *E2f1* KO retina has, as noted earlier, slightly fewer cells than the WT retina, although cell type proportions are unaffected (D. C. and R. B., unpublished data). Thus, marginally subnormal photopic responses in the *Rb/E2f1* DKO retina can be attributed to both a reduction of cone numbers in *E2f1* KO mice alone, and a “genuine” slight reduction in cone function attributable to *Rb* loss relative to WT. This slight effect may relate to a true differentiation defect in a subset of amacrine cells discussed below. This discussion should not obscure the major outcome that *E2f1* deletion recovers most of the ERG response. Thus, *E2f1* deletion not only rescues morphology but also both rod and cone system function in the *Rb* KO retina.

### Abnormal SAC Differentiation Independent of Cell Cycle and Survival Defects

**Figure 3:**
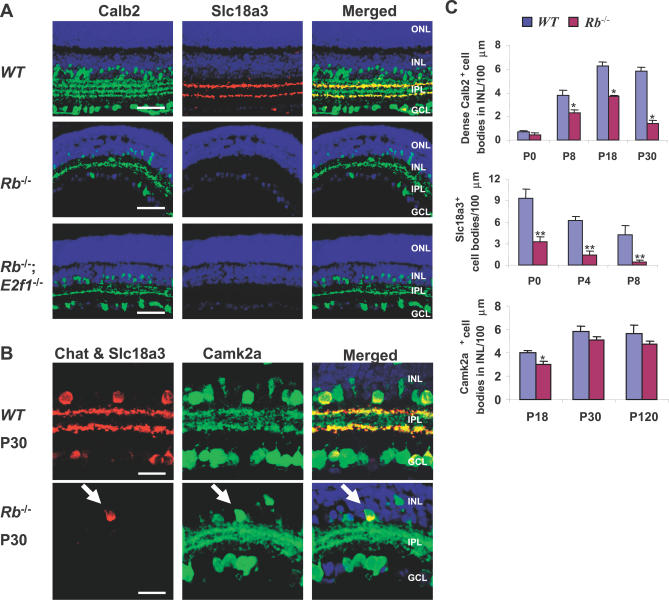
Differentiation Defects in *Rb* KO SACs (A) P18 horizontal sections of WT, *Rb* KO, and *Rb/E2f1* DKO retina were stained for nuclei (DAPI, blue), Calb2 (green), and Slc18a3 (red). (B) Confocal images of P30 horizontal sections of WT and *Rb* KO retina were stained for nuclei (DAPI, blue), Chat and Slc18a3 (both red), and Camk2a (green). In the *Rb* KO section, the red stain is Chat only, as Slc18a3 is missing (see [A]). (C) Quantification of dense Calb2^+^ cell bodies in the INL, total Slc18a3^+^ cell bodies, and Camk2a^+^ cell bodies in the INL. Error bars represent SD of measurements from three animals, and asterisks indicate significant differences between retinas of WT and the indicated genotypes (*, p <0.05; **; p <0.01; ANOVA and Tukey HSD test). Scale bars are 50 μm in (A) and 20 μm in (B).

ERGs primarily assess photoreceptor and bipolar cell function, but may miss differentiation defects in other cells. To test for subtle differences we stained the *Rb/E2f1* DKO retina with 43 markers (Table S1). Thirty-two proteins displayed identical patterns in WT, *E2f1* KO, and *Rb/E2f1* DKO retina (Table S1). The other 11 markers revealed a cell-cycle– and apoptosis-independent differentiation defect in SACs. We first studied Calb2 (calretinin), which marks a subset of amacrine and ganglion cell bodies as well as three tracks corresponding to their processes in the IPL (Figure 3A). Normal Calb2 staining was seen in the *E2f1* KO IPL (data not shown). However, only one Calb2^+^ track was evident in the *Rb* KO IPL, and this defect was not rescued in the *Rb/E2f1* DKO retina (Figure 3A). We quantified Calb2^+^ cell bodies in the *Rb* KO INL (corresponding to amacrine cell staining only) and observed a reduction from P8 onwards (Figures 3C and S6).

Of the three Calb2^+^ tracks in the IPL, the two outer tracks are from SACs, named after their extensive dendritic-tree-like morphology [37]. SACs are cholinergic, represent ~5.2% of amacrine neurons [38], and are critical for both direction selectivity [34,35] and spontaneous rhythmic activity that occurs during normal retinal development [36]. SACs in the INL synapse in the OFF layer of the IPL that responds to decreasing light, while displaced SACs in the GCL have processes that synapse in the nearby ON layer of the IPL that responds to increasing light (reviewed in [39]). Mature SAC processes stain specifically for Slc18a3 (vesicular acetyl choline transporter, VAChT) [37], and, significantly, this marker was absent in the peripheral *Rb* KO or *Rb/E2f1* DKO P18 retina (Figures 3A and S7B). Chat, expressed from the same locus, is also SAC specific, but marks both cell bodies and processes of mature SACs [37]. Chat was seen in fewer cells in the mature *Rb* KO retina, and was present in the soma but absent from processes (Figure 3B). We obtained similar results for Sv2c, a synaptic vesicle protein found in SACs [40]; Kcnc1b and Kcnc2, potassium channels expressed on SAC soma and dendrites as well as a very small number of ganglion cells [41]; gamma-aminobutyric acid (GABA), an inhibitory neurotransmitter present in about half of amacrine cells including SACs, as well as horizontal and some bipolar neurons [37]; and Calb1 (calbindin), which is expressed in many amacrine cells and labels SAC process faintly (Figure S7A and S7B; Table S1; and data not shown). Finally, we also examined the effect of *Rb* deletion on SAC differentiation using a *Chx10-Cre* transgene that is expressed in a mosaic pattern across the retina, generating patches of Cre-expressing cells [42]. Consistent with the mosaic deletion pattern, we observed markedly reduced Chat/Slc18a3 staining in the IPL of *Chx10-Cre;Rb^loxP/loxP^* retina compared to WT (Figure S7C). Together, these results suggest a role for Rb in SAC differentiation.

**Figure 4:**
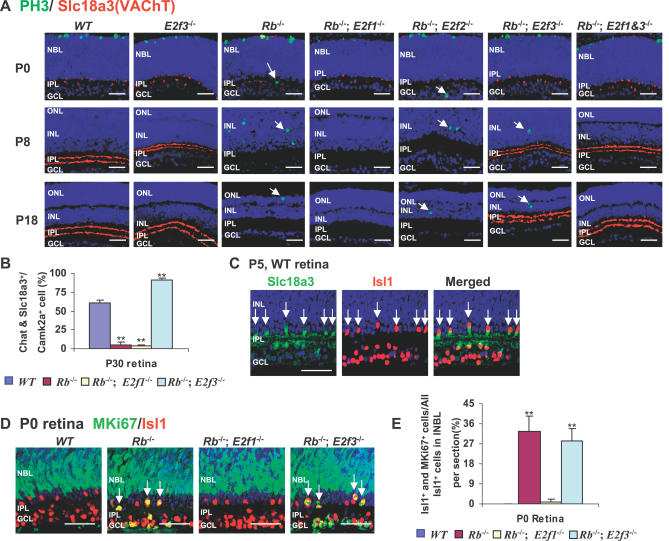
*E2f3* Loss Rescues Differentiation of *Rb* KO SACs (A) Horizontal retinal sections of the indicated ages and genotypes were stained for nuclei (DAPI, blue), mitosis marker PH3 (green), and Slc18a3 (red), which marks SAC soma at early stages and processes from ~P5 onwards. Arrows show mitotic PH3^+^ nuclei in *Rb* KO, *Rb/E2f2* DKO, and *Rb/E2f3* DKO retinas. *E2f1* loss rescues the ectopic mitosis and cell death defects, but not the SAC defect. *E2f2* loss has no effect. *E2f3* loss does not rescue the ectopic mitosis and cell loss defects, but rescues the SAC defect. Inactivating *E2f1* and *E2f3* together rescues the ectopic mitosis, cell death, and SAC defects. (B) The fraction of Camk2a^+^ cells that are Chat ^+^ and Slc18a3^+^ in the P30 retina. (C) Horizontal P5 retinal sections were stained for nuclei (DAPI, blue), Slc18a3 (green), and Isl1 (red). Arrows show double-labelled Isl1^+^/Slc18a3^+^ cells in the inner INL. (D) Horizontal P0 retinal sections of the indicated genotypes were stained for nuclei (DAPI, blue), cell division marker Mki67 (green), and Isl1 (red). Arrows show double-labelled dividing SACs. (E) The fraction of Isl1^+^ cells in the inner NBL (INBL) of P0 retinas that are dividing (Mki67^+^). Error bars represent SD of measurements from three animals, and asterisks indicate significant differences between retinas of WT and the indicated genotypes (*, p <0.05; **; p <0.01; ANOVA and Tukey HSD test). Scale bars in (A), (C), and (D) are 50 μm.

The above findings could indicate a defect in SAC specification, SAC survival, or the levels and/or transport of the markers described above. Camk2a marks both SACs and ganglion cells [37], but because ganglion cells are eliminated in the *Rb* KO retina, Camk2a is a specific SAC marker in this context. Importantly, Camk2a^+^ tracks and dendrites were present in both the WT and *Rb* KO retina (Figure 3B), and the number of Camk2a^+^ soma was similar in WT and *Rb* KO retina at P30 and beyond, although fewer cells stained in *Rb* KO retina at P18, suggesting a delay in its appearance (Figures 3C and S6B). Thus, Rb is not required for SAC survival or for process outgrowth, but rather it seems to regulate the expression and/or stability of Calb2, Calb1, Chat, Slc18a3, Sv2c, Kcnc1b, Kcnc2, and GABA in SACs, but leaves Camk2a expression virtually unaffected. The presence of Chat in some cell bodies but never in processes (Figure 3B) also suggests a transport defect. The developmental pattern of Slc18a3 expression also supported this notion. In mature WT SACs Slc18a3 is only in processes, but in early postnatal SACs, it is found in the cell body, and moves into emerging processes at approximately P4–P6. As noted above, Slc18a3 was absent at P18 in the *Rb* KO retina (Figure 3A); at P4 or P5 it was in cell bodies, yet was rarely present in *Rb* KO processes (Figures 4A and S6). Slc18a3 became virtually undetectable in *Rb* KO SACs by P8 (Figures 3C and S6C). These data suggest that Rb affects both the synthesis/stability and transport of SAC markers.

### Rb Regulates SAC Differentiation through E2f3

Rb binds more than 100 proteins [43] and in some non-neuronal cells, such as skeletal muscle, adipocytes, and bone, Rb is thought to bind and potentiate tissue-specific transcription factors that promote differentiation [31–33]. Thus, we expected that Rb might interact with retina-specific factors to facilitate SAC differentiation. A direct role for E2f in mediating Rb-dependent differentiation defects (independent of cell cycle or death defects) has to our knowledge not been described, but because E2f can regulate some differentiation genes [44–48], we first tested whether E2f2 or E2f3 might perturb *Rb* KO SAC maturation. At multiple time points, E2f1 deletion suppressed ectopic mitosis (PH3^+^ cells), but did not reverse the SAC defect, and *E2f2* deletion had no effect on either defect (Figure 4A). Remarkably, although E2f3 deletion did not reverse ectopic mitosis, it rescued Calb2, Slc18a3, Chat, GABA, Kcnc1b, Kcnc2, and Sv2c staining at multiple times (Figure 4A and data not shown). *Rb/E2f3* DKO SAC tracks were slightly more disordered than in WT retina, most likely because of the absence of synaptic partner cells, which are killed by E2f1. Indeed, this minor defect was rescued in the *Rb/E2f1/E2f3* triple knockout retina, where bipolar and ganglion cell death was rescued and SAC differentiation was restored (Figure 4A). *E2f3* deletion alone did not affect SAC differentiation (Figure 4A); thus, it is unleashed E2f3 activity that is detrimental, and the critical role for Rb is to inhibit E2f3.

We quantified the fraction of Camk2a^+^ SACs in different genotypes and found that 60% of WT P30 Camk2a^+^ cells expressed Chat and Slc18a3, which dropped to only 5.6% in the *Rb* KO retina, and remained low at 3.7% in the *Rb/E2f1* DKO retina, but rose to 91% in the *Rb/E2f3* DKO retina (Figure 4B). The latter fraction is higher than WT because ganglion cells, which normally make up ~40% of Camk2a^+^ cells, are killed by apoptosis.

To quantify the effect of different E2fs on ectopic division specifically in SACs, we exploited Isl1 (Islet1). This marker is expressed in both SACs and ganglion cells, thus Isl1^+^ cells in the INL are exclusively SACs [49]. We found that 98.2% ± 1.8% of Isl1^+^ cells in the forming inner INL at P5 were also Slc18a3^+^, confirming that Isl1 is an excellent SAC marker (Figure 4C). Moreover, Isl1, unlike Slc18a3, is nuclear, which facilitates scoring of Isl1^+^/Mki67^+^ cells. It is also expressed earlier than Slc18a3, permitting analysis of SACs soon after their birth at ~E15; thus, we could study retina at P0, a time when ectopic division is high in the inner retina and prior to Rb-independent cell cycle exit associated with terminal differentiation [2]. At P0, no WT Isl1^+^ cells in the inner neuroblastic layer (NBL) (which is the future INL) were dividing, but 57 ± 14 Isl1^+^/Mki67^+^ cells were detected in the *Rb* KO inner NBL (Figure 4D). Indeed, about one-third of all Isl1^+^ cells in the entire inner NBL were dividing in the *Rb* KO retina, or ~50% in the periphery where Cre is expressed (Figure 4E and data not shown). This defect was suppressed in the *Rb/E2f1* DKO retina, where we detected only 1 ± 1 dividing SAC, but not the *Rb/E2f3* DKO retina, where there were 53 ± 8 dividing SACs (Figure 4D and 4E). We observed similar effects at P0 with Calb2, which marks newborn SACs and other amacrine cells (data not shown). Thus, in *Rb* KO SACs, *E2f1* deletion suppresses ectopic division but not aberrant differentiation, whereas *E2f3* deletion suppresses aberrant differentiation but not ectopic division.

### Specific Expression of E2f3 in SACs and Other Subsets of CNS Neurons

**Figure 5:**
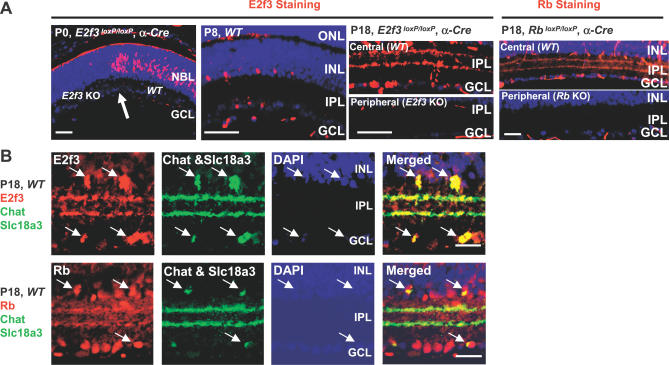
E2f3 and Rb Expression in SACs (A) Left panels: horizontal P0, P8, and P18 retinal sections of the indicated genotypes were stained for E2f3 (red) and DAPI (blue). The arrow indicates the junction between the *E2f3* null peripheral and WT central P0 retina. Note the absence of E2f3 protein in the peripheral *E2f3* KO RPCs at P0 and in peripheral inner retinal neurons at P18. Far right panel: P18 retinal sections of the indicated genotypes were stained for Rb (red) and DAPI (blue). Note the absence of Rb protein in the peripheral *Rb* KO inner retinal neurons. (B) WT P18 retinal sections were stained for nuclei (DAPI, blue), E2f3 (red) or Rb (red), and Chat plus Slc18a3 (green). Arrows indicate double-labelled soma. Note that the IPL processes are also double-labelled. Scale bars are 50 μm.

The unique effect of E2f3 in disrupting the differentiation of SACs but not other retinal neurons might be due to cell-type-specific expression or cell-type-specific activity of E2f3. Determining between these two possibilities is not easy, as E2f immunostaining in mouse tissues is problematic. We did not solve this issue for E2f1 or E2f2, but used a modified protocol [50] to successfully track E2f3 expression (Figure 5). At P0, E2f3 was detected in RPCs, consistent with a putative role in normal proliferation (Figure 5A). The signal was specific as it was absent in the *E2f3* KO peripheral retina (Figure 5A). As the retina differentiated and RPC division diminished, the number of E2f3^+^ cells also dropped, and by P8, when division is virtually over, only a subset of post-mitotic cells in the inner retina expressed E2f3 (Figure 5A). By P18, E2f3 was also detected in two tracks in the IPL (Figure 5A and 5B), reminiscent of SAC markers such as Chat and Slc18a3 (c.f. Figures 3 and 4). This cytoplasmic E2f3 staining was also specific, as it was absent in the *E2f3* KO peripheral retina of *α-Cre;E2f3^loxP/loxP^* mice (Figure 5A). Indeed, double labelling with E2f3 (red) and Chat plus Slc18a3 (green) confirmed that E2f3 is present in both SAC soma and dendrites (Figure 5B). Rb protein was also detected in the inner retina (Figure 5A), and showed a similar distribution as E2f3 in SACs (Figure 5B), and was also present in mature ganglion cells and Müller cells as reported [51]. Rb staining in SAC processes was specific as it was absent in the peripheral retina of *αCre;Rb^loxP/loxP^* mice (Figure 5A). These data suggest that Rb and E2f3 colocalize in SACs and that E2f3 triggers defects in SAC differentiation because it is specifically expressed in these retinal neurons.

We also found that E2f3 is present in a specific subset of mature neurons in various brain regions (data not shown). For example, in the P20 amygdala, E2f3 colocalized with the general neuronal markers Mtap2 and Mecp2 [52], but not with Calb2, which marks a subset of neurons, or with the glial marker Gfap (data not shown). Unlike in retinal SACs, E2f3 was not coexpressed in Chat^+^ or Slc18a3^+^ cholinergic neurons located in various regions of the brain and spinal cord (data not shown). In agreement, we could not detect defects in cholinergic *Rb* KO neurons in the developing forebrain, but other *Rb* KO neurons in this region showed differentiation defects that were rescued by deleting *E2f3* [53]. Together, these results suggest that the common mechanism by which Rb promotes neural differentiation is through E2f3 inhibition.

### Distinct Localization of E2f3 Isoforms

**Figure 6:**
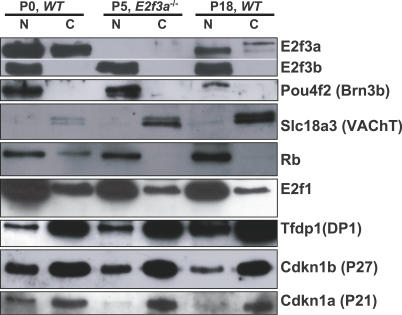
Subcellular Distribution of E2f3 Isoforms and Other Cell Cycle Proteins in the Developing Retina Nuclear and cytoplasmic extracts from an equivalent number of retinal cells from mice of the indicated genotypes and ages were analyzed by Western blotting to detect the indicated proteins. Lysate from *E2f3a^−/−^* mice was used as a control to confirm the location of E2f3a protein. C, cytoplasmic extracts; N, nuclear extracts.

**Figure 7:**
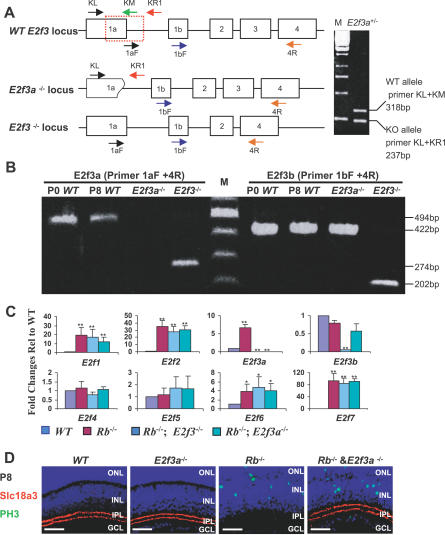
The E2f3a Isoform Drives the Differentiation Defect in *Rb* KO SACs (A) Schematic diagrams of the mouse WT, *E2f3a^−/−^*, and the Cre-recombined floxed *E2f3* loci (indicated here as *E2f3^−/−^* for simplicity). *E2f3a^−/−^* mice lack most of *E2f3* exon 1a and part of intron 1a (red dotted box). Arrows indicate PCR primers. Genotyping of an *E2f3a^+/−^* mouse is shown on the right. (B) RT-PCR detection of *E2f3a* and *E2f3b* mRNA in the retina. The sequences of primers are 1aF (5′-GCCTCTACACCACGCCACAAG-3′), 1bF (5′-CGGAAATGCCCTTACAGC-3′), and 4R (5′-CTCAGTCACTTCTTTGGACAG-3′). WT retina expresses both *E2f3a* and *E2f3b* mRNA. As expected, *E2f3a^−/−^* retina lacks *E2f3a* mRNA and still expresses *E2f3b* mRNA. *E2f3^−/−^* retina lacks full-length *E2f3a* and *E2f3b* mRNAs, and instead expresses a truncated mRNA lacking exon 3. (C) Real-time RT-PCR analysis of E2f genes in P8 retinas of the indicated genotypes. Error bars represent SD of measurements from three animals, and asterisks indicate a significant difference between WT and the indicated genotypes (*, p <0.05; **; p <0.01; ANOVA and Tukey HSD test). (D) Rescue of *Rb* KO SACs by *E2f3a* deletion. Horizontal retinal sections of the indicated genotypes and ages were stained for nuclei (DAPI, blue), M-phase (PH3, green), and the SAC marker Slc18a3 (red). *E2f3a* deletion does not suppress ectopic division, but rescues the SAC defect. Scale bars are 50 μm. M, molecular size marker.

As noted above, E2f3 and Rb staining in SACs was both nuclear and cytoplasmic (Figure 5A and 5B). The antibody that worked in immunostaining recognizes a C-terminal region and thus, does not distinguish a/b isoforms. To our knowledge, the subcellular location of E2f3 isoforms has not been determined in any cell type. To verify the dual locations of E2f3 and to determine which isoforms were present in retina, we analyzed nuclear and cytoplasmic fractions by Western blot at different times during development. Analysis with the pan-E2f3 antibody (sc-878, Santa Cruz Biotechnology) detected a 55-kD E2f3a species and a 40-kD E2f3b polypeptide (Figure 6). To confirm that the upper species in our retinal lysates was E2f3a, we exploited novel mice that lack *E2f3* exon 1a and thus express E2f3b exclusively (R. O. and G. L., unpublished data). The genotyping strategy is discussed in detail later and is outlined in Figure 7A. Western analysis confirmed that the upper band was absent in *E2f3a^−/−^* mice (Figures 6 and S8). Consistent with the drop in E2f3-expressing cells during WT retinal maturation (Figure 5A), the total amount of E2f3a was less at P18 compared to P0 (Figure 6). E2f3b was present in similar amounts at both time points. At P0 and P18, E2f3a was present in both nuclear and cytoplasmic fractions, but in marked contrast, E2f3b was exclusively nuclear at both times (Figure 6). Two closely migrating E2f3a bands were detected, more clearly evident at P18, of which the faster migrating species was dominant in nuclear and the slower species was dominant in cytoplasm (Figure 6). The identity of both as E2f3a species was confirmed by their absence in the P18 *E2f3a* KO retina (Figure S8). Analysis of Pou4f2, a nuclear transcription factor expressed in ganglion cells, showed that nuclear proteins had not contaminated the cytoplasmic fraction, and analysis of Slc18a3, a cytoplasmic SAC marker, confirmed that the reverse had also not occurred (Figure 6). These data show, to our knowledge for the first time, that E2f3a and E2f3b exhibit distinct patterns of subcellular distribution, and raise the possibility that E2f3a localization may be regulated by as yet unknown post-translational modifications.

We also examined the distribution of other cell cycle regulators during retinal development. Like E2f3a, Rb was present in both the WT cytoplasm and nucleus at P0, but at P18, when the levels of Rb had increased, it was primarily nuclear (Figure 6). A very faint cytoplasmic Rb signal was evident at P18, which is consistent with Rb staining of SAC processes (Figure 5B), and with the very small proportion of SACs in the retina [38]. E2f1 was also detected in both nuclear and cytoplasmic fractions, although unlike E2f3a it was predominantly nuclear both at P0 and P18 (Figure 6). The E2f dimerization partner, Tfdp1, which lacks a nuclear localization signal [54], was primarily cytoplasmic at both P0 and P18, and the Cdk inhibitors Cdkn1a and Cdkn1b showed a similar pattern of distribution (Figure 6). Thus, among the cell cycle regulators we examined, most showed bivalent distribution, and E2f3b was unusual in its solely nuclear compartmentalization.

### Rb Regulates SAC Differentiation through E2f3a

To test which E2f3 isoform is responsible for aberrant *Rb* KO SAC differentiation we exploited *E2f3a^−/−^* mice (Figure 7A). The genotyping strategy outlined in Figure 7A was used to distinguish the *E2f3a*, WT, and null alleles. Reverse transcriptase PCR (RT-PCR) confirmed the presence of both E2f3a and E2f3b RNA species in the developing WT retina, and the specific absence of E2f3a RNA in the *E2f3a^−/−^* retina (Figure 7B). E2f3a protein was absent in *E2f3a^−/−^* retinal lysate (Figures 6 and S8). Importantly, the levels of E2f3b message were similar in the *Rb* KO and *Rb/E2f3a* DKO retina, ruling out the possibility that any effects of E2f3a deletion we might observe were due to down-regulation of E2f3b (Figure 7C). Also, the levels of other E2fs were the same in the *Rb* KO, *Rb/E2f3* DKO, and *Rb/E2f3a* DKO retina, ruling out any cross-regulatory effects (Figure 7C) [55]. E2f3a can trigger cell cycle induction, but because SAC defects are not linked to cell cycle perturbation (Figures 3A and 4), and in view of the predominant association between E2f3b and Rb in quiescent cells [16,19], we suspected that E2f3b may perturb differentiation in *Rb* KO SACs. Unexpectedly, however, E2f3a deletion suppressed the *Rb* KO SAC defect (Figure 7D). Thus, separate from its role in cell cycle control, Rb regulation of E2f3a is critical to ensure proper neuronal differentiation.

## Discussion

### Rb Controls Retinal Cell Division and Death through E2f1

Work in the early 1990s showed that Rb loss triggers defects in neuronal cell cycle exit, survival, and differentiation [26–28]. Much of the death is an indirect consequence of probable hypoxia linked to placental defects [12–14]. However, targeted KO and chimeric studies reveal that Rb autonomously promotes cell cycle exit in newborn neurons, and is required for survival of a subset of neurons, particularly in the retina [2,3,13,14,56–59]. However, whether Rb also regulates differentiation is obscured by potentially indirect effects of ectopic division and death. Moreover, a mechanism though which Rb may regulate neuronal maturation has not been elucidated.

Here, deleting *E2f1* specifically rescued ectopic division and death in the *Rb* KO retina. Importantly, major *Rb*/*E2f1* DKO neurons differentiated normally, and ERGs revealed the rescue of rod- and cone-mediated function, implicating a regular signal flow from photoreceptors to bipolar and amacrine cells. Division and death genes were induced in *Rb* KO cells, and deleting *E2f1,* but not *E2f2* or *E2f3,* reversed these molecular events. E2f1 may also regulate differentiation targets, but whether this contributes to defects in retinal cell maturation is impossible to separate from potentially indirect consequences of deregulated division and death. In any case, it is clear that in most retinal cells, including photoreceptors [29], transcription factors that promote differentiation function independently of Rb.

We have also found that *E2f1* deletion rescues cell-autonomous ectopic division, death, and differentiation defects in sporadic *Rb* KO clones generated using a Cre retrovirus vector (M. P. and R. B., unpublished data). These data are consistent with the observation that E2f1 overexpression in newborn photoreceptors drives ectopic division and apoptosis [60], and add to the growing evidence indicating that E2f1 is the major, and perhaps only, member of the three activating E2fs required to induce apoptosis in *Rb* KO cells [10,15]. Thus, deregulated E2f1 activity in the retina, whether resulting from the inactivation of Rb or from overexpression, promotes unscheduled cell division and triggers apoptosis in susceptible RTCs. E2f1, rather than other E2fs, may be a potential target for novel therapeutics to prevent retinoblastoma in *RB1* ^+/−^ humans.

Our ERG studies revealed rescue of the *Rb* KO rod–bipolar system, and almost complete restoration of the cone–bipolar system following *E2f1* deletion. There was a slightly lower response in the *Rb/E2f1* DKO retina relative to the *E2f1* KO control retina. This difference might reflect a role for Rb in the development of cones, bipolar cells, or other cells that may contribute to the photopic ERG, including potentially SACs, which do have a serious defect in the *Rb/E2f1* DKO retina.

### Rb Controls SAC Differentiation through E2f3a

**Figure 8:**
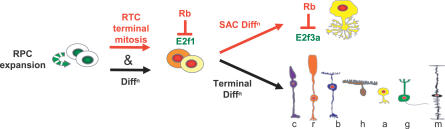
Rb Regulates Distinct Processes through E2f1 and E2f3a Red text and arrows indicate Rb-dependent events. Black text and arrows indicate events for which there is no direct evidence of Rb involvement. Rb does not appear to temper RPC expansion and is not required for differentiation of RPCs into RTCs, but is essential to couple RTC birth to terminal mitosis, thus locking them out of cycle. Rb performs this function by inhibiting E2f1. Rb is also required for SAC differentiation, and in this case, acts by inhibiting E2f3a. There is no direct evidence that Rb is required for terminal differentiation of other cell types. Colour codes and abbreviations as in Figure 1A.

Comprehensive marker analysis revealed that, in striking contrast to other retinal neurons, *E2f1* deletion did not suppress defects in *Rb* KO cholinergic SACs. Instead, we observed *E2f1*-independent defects in the synthesis and transport of a large cohort of SAC proteins. These data expand insight into the development of these important interneurons, but more critically, provide to our knowledge the first unambiguous evidence that Rb regulates neurogenesis beyond terminal mitosis. Rb binds more than 100 factors [43], and in several non-neuronal cells, such as skeletal muscle, adipocytes, and bone, it binds and potentiates tissue-specific transcription factors that promote differentiation [31–33]. The idea that Rb promotes muscle differentiation by potentiating Myod1 activity was contested [61], and other mechanisms proposed [62,63], but not involving E2f repression. Strikingly, however, we discovered that Rb promotes SAC differentiation through E2f3 (Figure 8).

Rb regulation of SAC differentiation through E2f3 was independent of its role in controlling division or death: *E2f3* deletion rescued *Rb* KO SAC defects but did not suppress aberrant proliferation or death, whereas *E2f1* deletion reversed abnormal proliferation and death but did not rescue SAC differentiation. Double labelling confirmed that E2f1 but not E2f3 deletion reversed *Rb* KO SAC division. Moreover, deleting *E2f1,* but not *E2f3,* reversed deregulated expression of cell cycle and apoptotic genes in the *Rb* KO retina. E2f3 is expressed in a subset of CNS neurons (this work) and drives specific cell-cycle–independent defects in *Rb* KO forebrain neurons [53]. Thus, E2f3 inhibition is the first, and may be the only, mechanism by which Rb participates directly in neuronal differentiation.

To further dissect the mechanism of action of Rb in SACs we determined the E2f3 isoform it targets to promote differentiation. E2f3b was the primary candidate, since Rb and E2f3b collaborate to repress targets in quiescent cells in vitro [19]. However, in the first work to our knowledge to examine the function of any E2f protein isoform in vivo, we made the surprising observation that Rb regulates SAC differentiation through E2f3a (Figure 8). Formally, we cannot exclude the possibility that deleting *E2f3b* might also rescue SAC differentiation, but definitive proof will require analysis of *E2f3b* null mice. Nevertheless, our data prove that Rb definitely regulates SAC differentiation through the activating E2f3 isoform.

### Distinct E2f3a and E2f3b Localization

The subcellular location of E2f isoforms has not to our knowledge been addressed before. E2f3a and E2f3b share 110 C-terminal amino acids that encode the NLS, DNA-binding, marked box, transactivation, and Rb-binding domains [16], yet they exhibit different subcellular distribution in developing retinal cells. E2f3a is both nuclear and cytoplasmic, but E2f3b is always nuclear. The unique 121- and six-residue N-termini of E2f3a and E2f3b, respectively, likely mediate this difference. This region in E2f1, E2f2, and E2f3a binds Ccna2, establishing a negative regulatory loop that deactivates E2fs in mid-late S-phase [64,65]. However, even E2f3b, which lacks this domain, binds and is regulated by Ccna2 [18], so the domain difference may not explain the unique distributions we observed. Rb family and Tfdp proteins can also determine E2f localization [20–22], and we found that a portion of both Rb and Tfdp1 proteins are cytoplasmic in retinal cells. Indeed, immunostaining revealed that Rb and E2f3 colocalize to SAC processes.

The nuclear localization of E2f3b contrasts with that of other repressive E2fs in differentiating muscle, where E2f5 switches from the nucleus to cytoplasm, while E2f4 remains in both compartments [23]. The distinct compartmentalization of E2f3a and E2f3b in the retina suggests temporally and functionally distinct activities. Rb distribution matches that of E2f3a, consistent with its critical role in supporting SAC differentiation through E2f3a.

### Ectopic Division and Differentiation

Rb is critical to ensure that many types of terminally differentiating cells leave the cell cycle (e.g., neurons, gut and skin epithelia, muscle, and lens fibres) (reviewed in [66]). Early overexpression studies in vitro suggested Rb might temper expansion of cycling cells, but KO studies in vivo indicate that its major role is to block division in terminally differentiating cells. In its absence, many (but clearly not all) aspects of differentiation go ahead relatively unperturbed. In the retina, differentiating transition cells are born in the absence of Rb, migrate to the correct layer, and express appropriate markers ([2] and this work). In brain, *Rb* KO neurons migrate away from the ventricular zone and switch on Tubb3 (βIII-tubulin), but continue to incorporate BrdU [13], and in gut epithelia, differentiated enterocytes migrate up the villi and activate expression of serotonin, yet continue to incorporate BrdU [67]. In the case of SACs, the differentiation defects we observed (e.g., loss of Slc18a3 and Chat) were not due to aberrant division, but it is possible there are other problems with these cells that are caused by ectopic division. Nevertheless, it is clear that many aspects of differentiation in multiple cell types are compatible with ectopic division. However, division of terminally differentiating cells is dangerous, since it may facilitate transformation, as is the case in retinoblastoma (reviewed in [66]).

### How Does E2f3a Perturb SAC Differentiation?

E2f3a could disrupt SAC differentiation through its well known role as a transcriptional activator, or, in view of the discovery that it is partially cytoplasmic, E2f3a may affect processes other than gene regulation. Both scenarios are feasible since E2fs regulate differentiation genes [44–48], and cell cycle regulators, such as Cdkn1b, have cytoplasmic activities that influence differentiation [68,69]. Many transcription factors shuttle between nucleus and cytoplasm during neurogenesis (e.g., [70] and references therein). It may be difficult to identify E2f3a-specific target genes or cytoplasmic proteins in SACs since these neurons are a small proportion (<1%) of the total retina and only ~5.2% of amacrine neurons [38].

### Do E2fs Mediate All Rb Functions?

Others have suggested that Rb promotes differentiation in non-neuronal cells through E2f-independent means [31–33]. However, these studies did not assess whether these cell types differentiate normally if Rb is deleted along with one or more E2f family members. One study reported that Rb mutants that do not bind E2f still induce differentiation [30]. However, the binding assays were performed in solution, and we have found that several of these mutants do bind E2f, albeit weakly, on chromatin (T. Yu and R. B., unpublished data). It is possible that Rb-mediated potentiation of tissue-specific transcription factors may, at least in some cases, be a redundant activity, and that the only critical Rb function is to inhibit E2f. Our study is the first to our knowledge to assess comprehensively whether *Rb* KO cells can differentiate in the absence of different E2fs. In light of our findings, it will be important to reassess differentiation defects in other *Rb* KO tissues in the absence of individual and combined activating E2f family members.

## Materials and Methods

### Mouse strains and genotyping.

Mice were treated according to institutional and national guidelines. *α-Cre* mice (P. Gruss), *Chx10-Cre* mice (C. Cepko), *Rb^loxP/loxP^* mice (A. Berns), *E2f1^–/–^* mice, *E2f2^–/–^* mice, *E2f3^loxP/loxP^* mice, and *E2f3a^−/−^* mice were maintained on a mixed (NMRI × C57/Bl × FVB/N × 129sv) background. A detailed description of *E2f3a^−/−^* mice will be published elsewhere. Mice of different genotypes were compared within the same litter and across a minimum of three litters. We have not noted any phenotypic differences in separate litters. Genotyping was performed as before [2,5], and the primers used for genotyping *E2f3a^−/−^* mice were E2f3a KL (5′-CTCCAGACCCCCGATTATTT-3′), E2f3a KR1 (5′-TCCAGTGCACTACTCCCTCC-3′), and E2f3a KM (5′-GCTAGCAGTGCCCTTTTGTC-3′).

### Histology, immunofluorescence, and measurements.

Eyeballs were fixed in 4% paraformaldehyde for 1 h at 4 °C, embedded in OCT (TissueTek 4583, Sakura, http://www.sakuraeu.com), frozen on dry ice, and cut into 12-μm sections on Superfrost plus slides (VWR, http://www.vwr.com). For S-phase analysis, BrdU (100 μg/g of body weight) was injected intraperitoneally 2 h prior to sacrifice. BrdU^+^ cells were detected using a biotin-conjugated sheep polyclonal antibody (1:500, Maine Biotechnology Services, http://www.mainebiotechnology.com). All other antibodies are described in Table S1. For E2f3, Mki67, and Rb staining, antigen retrieval was performed by boiling sections in citric acid solution for 15 min according to Ino [50], except on frozen sections. TUNEL was performed as described [13]. Briefly, sections were incubated for 1 h at 37 °C with 75 μl of mixture solution consisting of 0.5 μl of terminal deoxynucleotide transferase, 1 μl of biotin-16-dUTP, 7.5 μl of CoCl_2_, 15 μl of 5× terminal deoxynucleotide transferase buffer, and 51 μl of distilled water. After three washes in 4× SSC buffer, sections were incubated with Alexa 488– or Alexa 568−streptavidin (1:1,000; Molecular Probes, http://probes.invitrogen.com) for 1 h at room temperature. Primary antibodies or labelled cells were visualized using donkey anti-mouse Alexa 488 or Alexa 568, donkey anti-rabbit Alexa 488 or Alexa 568, donkey anti-goat Alexa 488 or Alexa 568, and streptavidin Alexa 488 or Alexa 568 (1:1,000; Molecular Probes). Nuclei were counter-stained with 4,6-diamidino-2-phenyindole (DAPI; Sigma, http://www.sigmaaldrich.com). Labelled cells were visualized using a Zeiss (http://www.zeiss.com) Axioplan-2 microscope with Plan Neofluar objectives and images captured with a Zeiss AxionCam camera. For double-labelled samples, confocal images were obtained with a Zeiss LSM 5.0 laser scanning microscope.

The retina was separated into three bins by dividing the ventricular edge of the retina into equal parts and extending a line to the vitreal edge [2]. Bin 1 contains only cells that expressed Cre as progenitors; bin 3 is at central retina and contains cells derived from progenitors that did not express Cre. For cell counts or thickness measurement we used a region 0–100 μm peripheral to the boundary separating bins 1 and 2. Measurements were performed on an Axioplan-2 microscope using Axiovison software. Quantification of S-phase, M-phase, and apoptotic cells was performed on horizontal sections that included the optic nerve. Quantification of differentiated cell types was performed using horizontal sections at equal distances from the optic nerve. A minimum of three sections per eye and three eyes from different litters were counted.

### RNA extraction, reverse transcription, and PCR.

Total RNA was isolated from dissected peripheral retina using TRIzol reagent (Invitrogen, http://www.invitrogen.com) followed by digestion with RNase-free DNase (DNA-*free*, Ambion, http://www.ambion.com) to remove DNA contamination. First-strand cDNA was synthesized from 0.2–0.5 μg of total RNA using the SuperScript II first-strand synthesis system (Invitrogen). PCR primers are listed in Table S2. Real-time quantitative PCR was performed using an Applied Biosystems (http://appliedbiosystems.com) PRISM 7900HT. Tests were run in duplicate on three separate biological samples with SYBR Green PCR Master Mix (Applied Biosystems) exactly as we described previously [71]. Briefly, master stocks were prepared such that each 10-μl reaction contained 5 μl of SYBR Green PCR Master Mix, 0.1 μl of each forward and reverse primer (stock 50 μM), 0.8 μl of blue H_2_O (0.73% Blue Food Colour; McCormick, http://www.mccormick.com), 2 μl of diluted cDNA template, and 2 μl of yellow H_2_O (0.73% Yellow Food Colour). PCR consisted of 40 cycles of denaturation at 95 °C for 15 s and annealing and extension at 55 °C for 30 s. An additional cycle (95 °C, 15 s, 60 °C) generated a dissociation curve to confirm a single product. The cycle quantity required to reach a threshold in the linear range was determined and compared to a standard curve for each primer set generated by five 3-fold dilutions of genomic DNA or cDNA samples of known concentration. Values obtained for test RNAs were normalized to Hprt1 mRNA levels.

### Western blots.

Mouse retinas were homogenized by passing them through a 30-gauge BD 9 http://www.bd.com) needle 5–10 times in 1× PBS solution. Nuclear and cytoplasmic proteins were extracted using the NE-PER Nuclear and Cytoplasmic Extraction Kit (Product# 78833, Pierce Biotechnology, http://www.piercenet.com). Proteins were separated by 10% SDS-PAGE and transferred to nitrocellulose. After blocking overnight at 4 °C in 5% skim milk, membranes were incubated in the primary antibody for 2 h at room temperature. After three 10-min washes in TPBS (100 mM Na_2_HPO_4_, 100 mM NaH_2_PO_4_, 0.5 N NaCl, 0.1% Tween-20), membranes were incubated for 30 min at room temperature in the secondary horseradish peroxidase-conjugated antibody (Jackson ImmunoResearch Laboratories, http://www.jacksonimmuno.com). Blots were developed using the ECL-Plus chemiluminescent detection system (Amersham Pharmacia Biotech, http://www.pharmacia.ca), according to the manufacturer's instructions.

The following primary antibodies were used: E2f-1 (SC-193), E2f-3 (SC-878), Cdkn1a (p21, SC-471), Cdkn1b (p27, SC-528), Pou4f2 (Brn3b, SC-6062), and Tfdp1 (Dp1, SC-610) from Santa Cruz Biotechnology (http://www.scbt.com), pRB (554136) from BD Science-Pharmingen (http://www.bdbiosciences.com), and Slc18a3 (VAChT, G448A) from Promega (http://www.promega.com).

### Electroretinography.

ERGs were recorded from dark-adapted mice as described [72]. Briefly, mice were dark-adapted overnight and anaesthetized by subcutaneous injection of ketamine (66.7 mg/kg body weight) and xylazine (11.7 mg/kg body weight). The pupils were dilated and single-flash ERG recordings were obtained under dark-adapted (scotopic) and light-adapted (photopic) conditions. Light adaptation was accomplished with a background illumination of 30 candela (cd) per square meter starting 10 min before recording. Single white-flash stimulation ranged from 10^−4^ to 25 cd·s/m^2^, divided into ten steps of 0.5 and 1 log cd·s/m^2^. Ten responses were averaged with an inter-stimulus interval of either 5 s (for 10^−4^, 10^−3^, 10^−2^, 3 × 10^−2^, 10^−1^, and 3 × 10^−1^ cd·s/m^2^) or 17 s (for 1, 3, 10, and 25 cd·s/m^2^). Band-pass filter cut-off frequencies were 0.1 and 3,000 Hz.

### Statistics.

Different genotypes were evaluated using analysis of variance (ANOVA) followed by the Tukey honestly significant difference (HSD) test or Fisher test (XLSTAT program, http://www.xlstat.com).

## Supporting Information

Figure S1 Deleting *E2f1,* but Not *E2f2* or *E2f3,* Rescues Ectopic Division and Cell Death in P0 and P18 *Rb* KO Retina Horizontal sections of the indicated genotypes and ages were stained for nuclei (DAPI, blue), and (A) S-phase (anti-BrdU, red) or (B) apoptosis (TUNEL, red). In *Rb* ^−/−^ retinas, BrdU^+^ cells extend beyond the normal boundaries at P0 (arrows), and ectopic DNA synthesis continues in multiple layers at later stages. Scale bar is 50 μm. The NBL is where dividing RPCs are located.
(815 KB PDF)

Figure S2 Deleting *E2f1* Rescues Ectopic Division and Apoptosis in the Embryonic *Rb* KO Retina Horizontal sections of the indicated genotypes and ages (E14 and E16, the period during which SACs are born) were stained for nuclei (DAPI, blue), and either S-phase (upper two panels, anti-BrdU, red) or apoptosis (lower two panels, TUNEL, red). In *Rb* ^−/−^ retinas, BrdU^+^ and TUNEL^+^ cells can be seen in the inner retina (arrows). Inactivation of *E2f1* rescued these defects. Scale bar is 50 μm. The NBL is where dividing RPCs are located.
(754 KB PDF)

Figure S3 Deleting *E2f1,* but Not *E2f2* or *E2f3,* Rescues Ectopic Mitosis in the *Rb* KO Retina (A) Horizontal retinal sections of the indicated genotypes and ages were stained for nuclei (DAPI, blue) and M-phase (anti-PH3, red). Scale bar is 50 μm.
(B) Quantification of all PH3^+^ cells.
(C) Quantification of ectopic PH3^+^ cells.
Error bars represent standard deviation (SD), and asterisks indicate significant difference between retina of WT and indicated genotypes (*, p <0.05; **, p <0.01; ANOVA and Tukey HSD test).
(628 KB PDF)

Figure S4 Deleting *E2f2* or *E2f3* Does Not Rescue Ganglion, Rod, or Bipolar Cell Death in the *Rb* KO Retina (A) Horizontal retinal sections from mice of the indicated ages and genotypes were stained for nuclei (DAPI, blue) and markers that detect ganglion cells (Pou4f2, red), rods and cones (Sag [rod arrestin], green), and rod bipolar cells (Prkca, green). Scale bar is 50 μm.
(B) Quantification of total ganglion (Pou4f2^+^) cells.
(C) Quantification of total rod bipolar (Prkca^+^) cells.
(D) Thickness of the ONL, which represents the number of rods.
Error bars represent SD, and asterisks indicate significant difference between retina of WT and indicated genotypes (**, p <0.01; ANOVA and Tukey HSD test).
(488 KB PDF)

Figure S5 *E2f1* Deletion Rescues *α-Cre;Rb^loxP/loxP^* Retinal Function ERGs were recorded from the indicated genotypes under light adapted (photopic) conditions.
(A) Intensity series.
(B) The b-wave amplitudes as a function of the logarithm of the flash intensity.
(383 KB PDF)

Figure S6 Differentiation Defects in *Rb* KO SACs Horizontal retinal sections of indicated genotypes and ages were stained for nuclei (DAPI, blue) and Calb2 ([A], red; only densely stained cells were counted for Figure 3C), Camk2a ([B], green), and Slc18a3 ([C], red). Scale bars are 50 μm.
(564 KB PDF)

Figure S7 GABA Neurotransmitter in the *Rb* KO Retina and Abnormal SACs in *Chx10-Cre;Rb^loxP/loxP^* Retina Horizontal sections of the indicated genotypes and ages of retina were stained for nuclei (DAPI, blue), and (A and B) GABA (red) and Slc18a3 (green) or (C) Chat and Slc18a3 (red).
(A) In P18 WT retina, GABA labelled four IPL tracks, of which the two inner tracks co-stained with Slc18a3. The latter tracks disappeared in the *Rb* KO retina, and were rescued by *E2f3* KO but not *E2f1* KO.
(B) At the boundary of the WT (central) and *Rb* KO area (peripheral retina) the inner GABA^+^ SAC tracks can be seen disappearing towards the periphery (left).
(C) Slc18a3 staining in the IPL of *Chx10-Cre;Rb^loxP/loxP^* retina is consistent with the mosaic pattern of *Rb* inactivation.
Scale bars are 50 μm.
(633 KB PDF)

Figure S8 Subcellular Distribution of E2f3a Isoform in the Developing Retina Nuclear and cytoplasmic extracts from an equivalent number of retinal cells from mice of the indicated genotypes and ages were analyzed by Western blotting to detect the E2f3a protein. Lysates from *E2f3a^−/−^* mice of matched ages were used as a control to confirm the location of E2f3a protein. C, cytoplasmic extracts; N, nuclear extracts.
(115 KB PDF)

Table S1 List of Antibodies and Marker Patterns in *Rb/E2f1* DKO SACs (97 KB DOC)

Table S2 Real-Time RT-PCR Primers (49 KB DOC)

### Accession Numbers

The GenBank (http://www.ncbi.nlm.nih.gov/genbank) accession numbers for the major genes and gene products discussed in this paper are Camk2a (NM_009792), Chat (NM_009891), E2f1 (NM_007891), E2f2 (NM_177733), E2f3 (NM_010093), Rb (NM_009029), and Slc18a3 (NM_021712).

## Abbreviations

ANOVA: analysis of variance
BrdU: bromodeoxyuridine
cd: candela
CNS: central nervous system
DAPI: 4,6-diamidino-2-phenyindole
DKO: double knockout
E[number]: embryonic day [number]
E2f: E2f transcription factor
ERG: electroretinogram
HSD: honestly significant difference
GABA: gamma-aminobutyric acid
GCL: ganglion cell layer
INL: inner nuclear layer
IPL: inner plexiform layer
KO: knockout
NBL: neuroblastic layer
ONL: outer nuclear layer
OPL: outer plexiform layer
P[number]: postnatal day [number]
PH3: phosphohistone 3
Rb: retinoblastoma protein
RPC: retinal progenitor cell
RTC: retinal transition cell
RT-PCR: reverse transcriptase PCR
SAC: starburst amacrine cell
SD: standard deviation
TUNEL: terminal dUTP nick-end labelling
WT: wild-type
