# Supplementary figures and images for: Rb-Mediated Neuronal Differentiation through Cell-Cycle–Independent Regulation of E2f3a
Source: PLoS Biol. 2007 Jul 3;5(7):e179. doi: 10.1371/journal.pbio.0050179 (PMC1914394; doi:10.1371/journal.pbio.0050179)

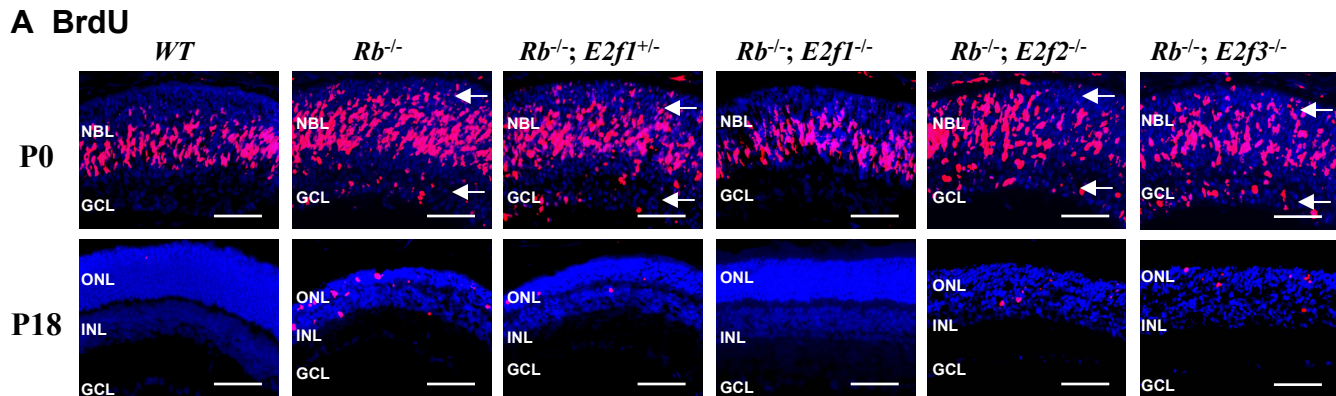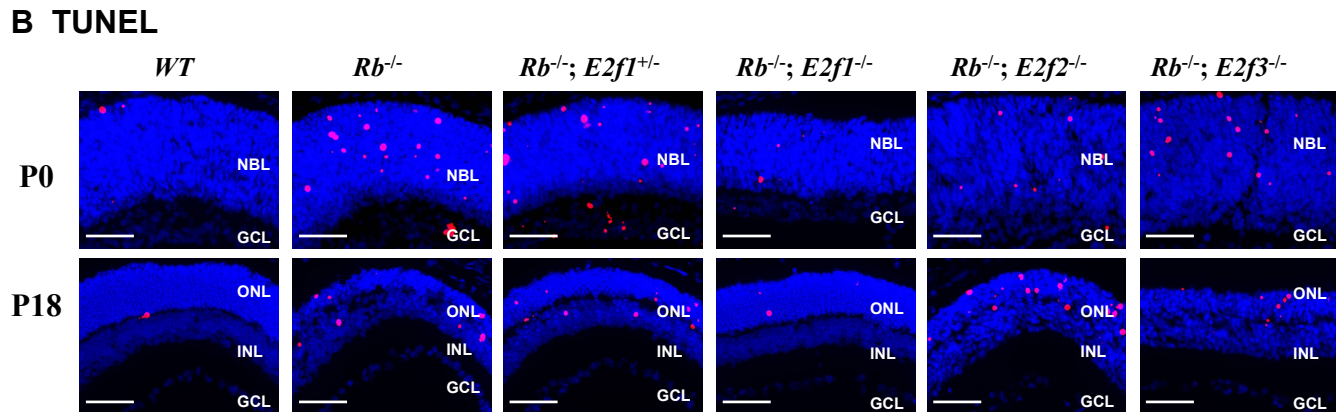

Supplement: Figure S1 — Horizontal sections of the indicated genotypes and ages were stained for nuclei (DAPI, blue), and (A) S-phase (anti-BrdU, red) or (B) apoptosis (TUNEL, red). In Rb −/− retinas, BrdU+ cells extend beyond the normal boundaries at P0 (arrows), and ectopic DNA synthesis continues in multiple layers at later stages. Scale bar is 50 μm. The NBL is where dividing RPCs are located. (815 KB PDF) [file pbio.0050179.sg001.pdf]

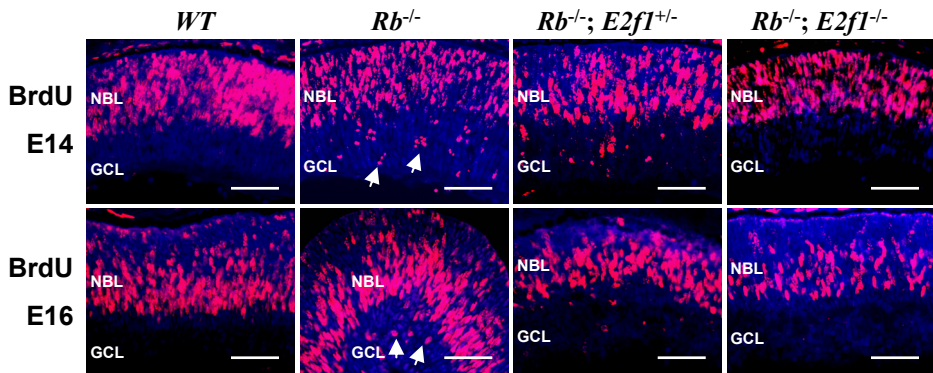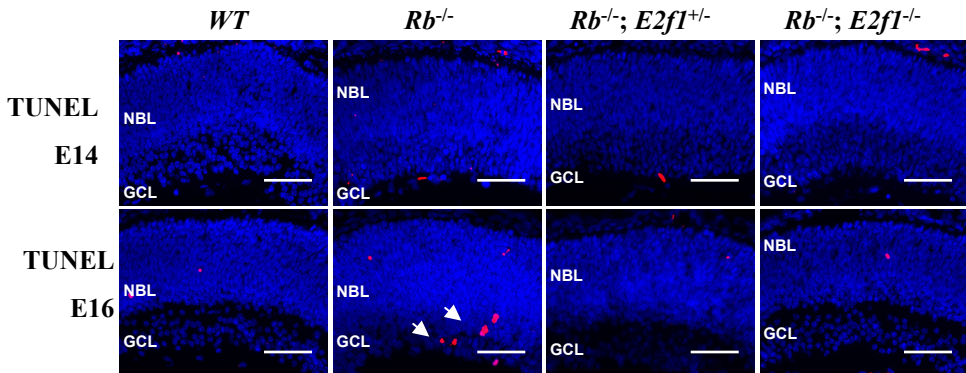

Supplement: Figure S2 — Horizontal sections of the indicated genotypes and ages (E14 and E16, the period during which SACs are born) were stained for nuclei (DAPI, blue), and either S-phase (upper two panels, anti-BrdU, red) or apoptosis (lower two panels, TUNEL, red). In Rb −/− retinas, BrdU+ and TUNEL+ cells can be seen in the inner retina (arrows). Inactivation of E2f1 rescued these defects. Scale bar is 50 μm. The NBL is where dividing RPCs are located. (754 KB PDF) [file pbio.0050179.sg002.pdf]

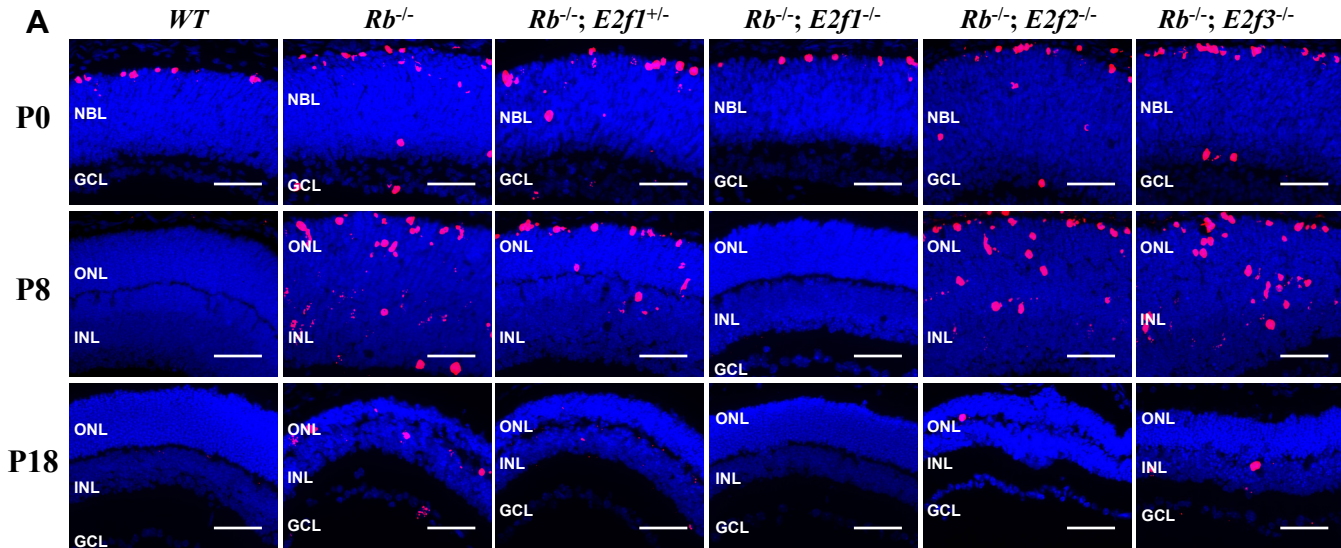

**B**

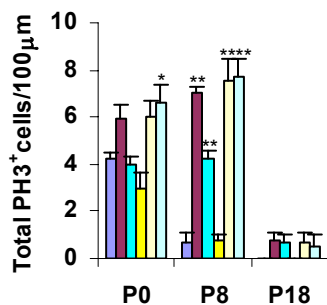

**C**

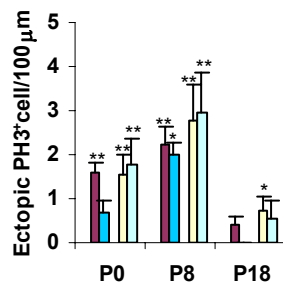

■ *WT*   
 ■ *Rb*<sup>-/-</sup>   
 ■ *Rb*<sup>-/-</sup>; *E2f1*<sup>+/-</sup>   
 ■ *Rb*<sup>-/-</sup>; *E2f1*<sup>-/-</sup>   
 ■ *Rb*<sup>-/-</sup>; *E2f2*<sup>-/-</sup>   
 ■ *Rb*<sup>-/-</sup>; *E2f3*<sup>-/-</sup>

Supplement: Figure S3 — (A) Horizontal retinal sections of the indicated genotypes and ages were stained for nuclei (DAPI, blue) and M-phase (anti-PH3, red). Scale bar is 50 μm. (B) Quantification of all PH3+ cells. (C) Quantification of ectopic PH3+ cells. Error bars represent standard deviation (SD), and asterisks indicate significant difference between retina of WT and indicated genotypes (*, p <0.05; **, p <0.01; ANOVA and Tukey HSD test). (628 KB PDF) [file pbio.0050179.sg003.pdf]

Figure S4

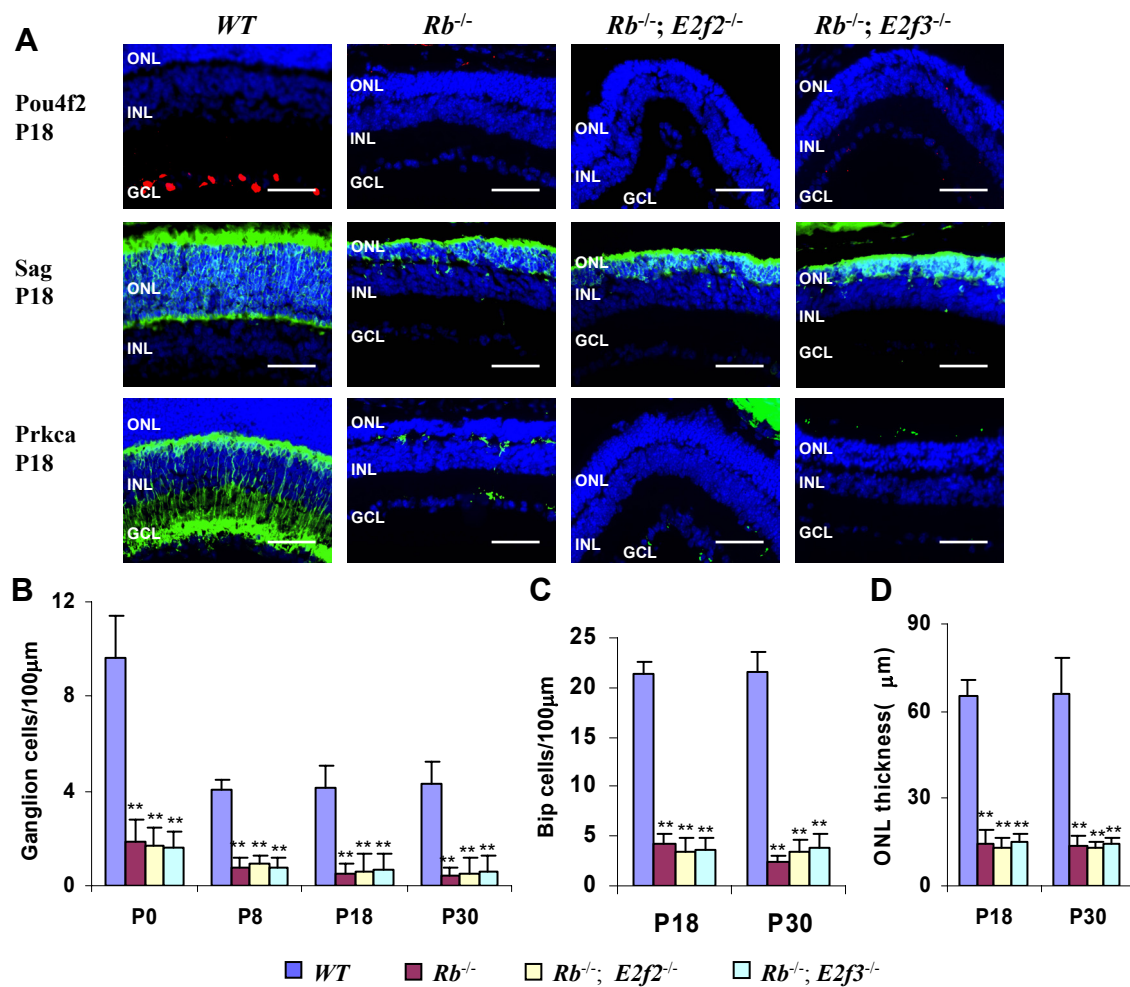

Supplement: Figure S4 — (A) Horizontal retinal sections from mice of the indicated ages and genotypes were stained for nuclei (DAPI, blue) and markers that detect ganglion cells (Pou4f2, red), rods and cones (Sag [rod arrestin], green), and rod bipolar cells (Prkca, green). Scale bar is 50 μm. (B) Quantification of total ganglion (Pou4f2+) cells. (C) Quantification of total rod bipolar (Prkca+) cells. (D) Thickness of the ONL, which represents the number of rods. Error bars represent SD, and asterisks indicate significant difference between retina of WT and indicated genotypes (**, p <0.01; ANOVA and Tukey HSD test). (488 KB PDF) [file pbio.0050179.sg004.pdf]

**A****Photopic**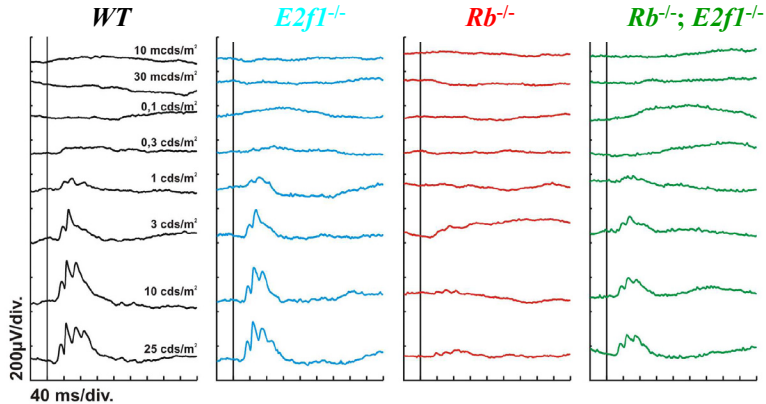**B****Photopic**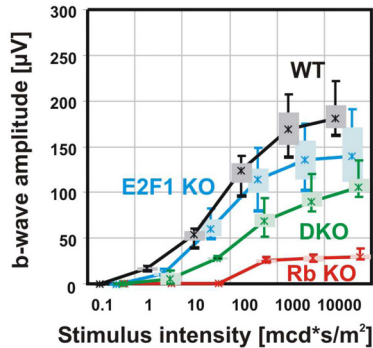

Supplement: Figure S5 — ERGs were recorded from the indicated genotypes under light adapted (photopic) conditions. (A) Intensity series. (B) The b-wave amplitudes as a function of the logarithm of the flash intensity. (383 KB PDF) [file pbio.0050179.sg005.pdf]

**Figure S6**

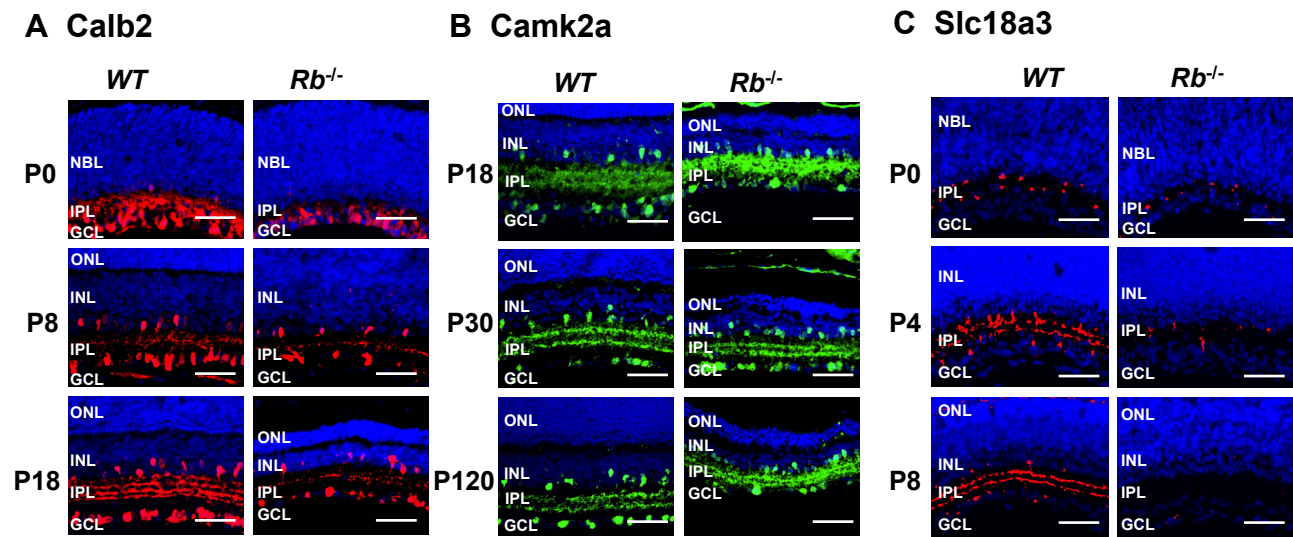

Supplement: Figure S6 — Horizontal retinal sections of indicated genotypes and ages were stained for nuclei (DAPI, blue) and Calb2 ([A], red; only densely stained cells were counted for Figure 3C), Camk2a ([B], green), and Slc18a3 ([C], red). Scale bars are 50 μm. (564 KB PDF) [file pbio.0050179.sg006.jpg]

Figure S7

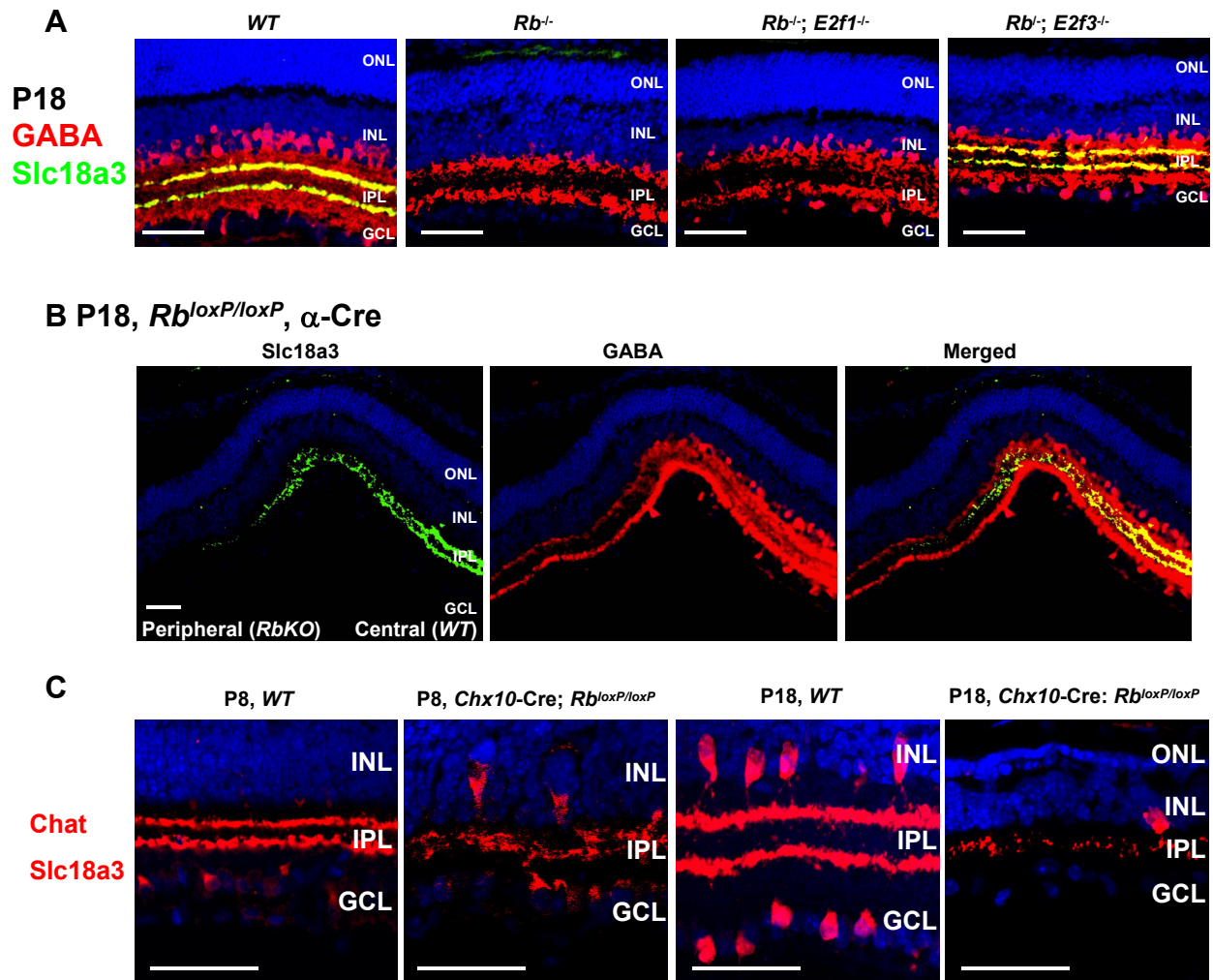

Supplement: Figure S7 — Horizontal sections of the indicated genotypes and ages of retina were stained for nuclei (DAPI, blue), and (A and B) GABA (red) and Slc18a3 (green) or (C) Chat and Slc18a3 (red). (A) In P18 WT retina, GABA labelled four IPL tracks, of which the two inner tracks co-stained with Slc18a3. The latter tracks disappeared in the Rb KO retina, and were rescued by E2f3 KO but not E2f1 KO. (B) At the boundary of the WT (central) and Rb KO area (peripheral retina) the inner GABA+ SAC tracks can be seen disappearing towards the periphery (left). (C) Slc18a3 staining in the IPL of Chx10-Cre;RbloxP/loxP retina is consistent with the mosaic pattern of Rb inactivation. Scale bars are 50 μm. (633 KB PDF) [file pbio.0050179.sg007.pdf]

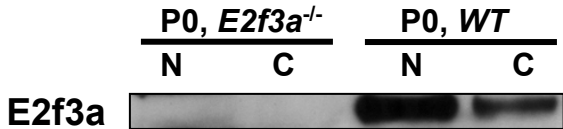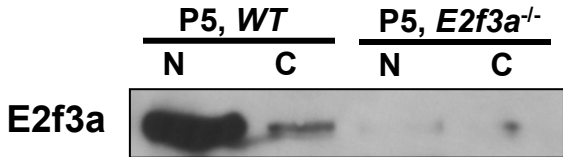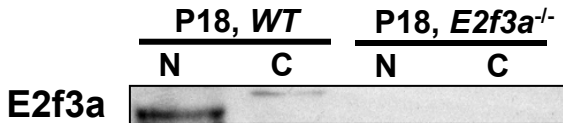

Supplement: Figure S8 — Nuclear and cytoplasmic extracts from an equivalent number of retinal cells from mice of the indicated genotypes and ages were analyzed by Western blotting to detect the E2f3a protein. Lysates from E2f3a−/− mice of matched ages were used as a control to confirm the location of E2f3a protein. C, cytoplasmic extracts; N, nuclear extracts. (115 KB PDF) [file pbio.0050179.sg008.pdf]
