# Supplementary material for: Rb-Mediated Neuronal Differentiation through Cell-Cycle–Independent Regulation of E2f3a
Source: PLoS Biol. 2007 Jul 3;5(7):e179. doi: 10.1371/journal.pbio.0050179 (PMC1914394; doi:10.1371/journal.pbio.0050179)
Supplement: Table S1 — (97 KB DOC) [file pbio.0050179.st001.doc]

**Table S1. List of antibodies and marker patterns in *Rb/E2f1* DKO SACs.**

| Antibody | Cell types/Structures | Working conc. | Species | Source | *Rb/E2f1* DKO* |
| --- | --- | --- | --- | --- | --- |
| Arr3 (Cone arrestin) | Mature cones | 1:2000 | Rabbit polyclonal | CM Craft and X Zhu, U South California | N |
| Atp2b1 (PMCA1) | Subset of bipolar cells | 1:100 | Rabbit polyclonal | Abcam AB3528 | N |
| Brdu | Proliferative cells (S phase) | 1:500 | Sheep polyclonal | Maine Biotechnology Service PAB105B | N |
| Cabp5 | Rod and Cone Bipolars | 1:500 | Rabbit polyclonal | F Haeseleer, U Washington | N |
| Calb1 (Calbindin) | Horizontal, subset of amacrines (faint in WT SAC processes). | 1:2000 | Mouse monoclonal | Sigma C9848 | A |
| Calb2 (Calretinin) | Subset of amacrine (incl. SACs) and ganglion cells | 1:2000 | Rabbit polyclonal | Chemicon AB5054 | A |
| Camk2a | SACs, ganglion cells | 1:200 | Mouse monoclonal | Chemicon MAB8699 | A-delay |
| Ccnd3 (Cyclin D3) | Müller glia | 1: 200 | Mouse monoclonal | Santa Cruz SC-6283 | N |
| Cdkn1b (p27^Kip1^) | Müller glia | 1:100 | Rabbit polyclonal | Santa Cruz SC-528 | N |
| Cdkn1c (p57^Kip2^) | Amacrine subtypes | 1:50 | Rabbit polyclonal | Santa Cruz SC-8298 | N |
| Chat | SACs | 1:100 | Rabbit polyclonal | Chemicon AB5042 | A |
| Chx10 | RPCs, transition and mature bipolar cells | 1:500 | Rabbit polyclonal | R. McInnes and RL Chow, U Toronto | N |
| E2f3 | Dividing RPCs, SACs | 1:100 | Mouse monoclonal | Upstate 05-551 | A |
| GABA | GABA-ergic amacrines (which includes SACs) | 1:2000 | Rabbit polyclonal | Chemicon AB131 | A |
| Gfap | Astrocytes and activated mature Müller glia | 1:1000 | Rabbit polyclonal | Sigma G9269 | N |
| Isl1 | SACs, ganglions and bipolars | 1:500 | Rabbit polyclonal | T Edlund, U Umea, Sweden | A |
| Kcnc1b (Kv3.1b) | SACs | 1:100 | Rabbit polyclonal | Alomone labs APC-014 | A |
| Kcnc2 (Kv3.2) | SACs | 1:100 | Rabbit polyclonal | Alomone labs APC-011 | A |
| Mecp2 | All neurons except rods | 1:500 | Rabbit polyclonal | Upstate #07-013 | N |
| MKi67 | M and S phase | 1:200 | Mouse monoclonal | BD science Pharmingen 550609 | N |
| Mtap2 (Map2) | Neurons | 1:200 | Rabbit polyclonal | Chemicon AB5622 | N |
| Nos1 | Amacrine subtypes | 1:100 | Rabbit polyclonal | Santa Cruz SC-648 | N |
| Opn1mw (M opsin) | M cones | 1:2000 | Rabbit polyclonal | CM Craft and X Zhu, U South California | N |
| Opn1sw (S Opsin) | S cones | 1:2000 | Rabbit polyclonal | CM Craft and X Zhu, U South California | N |
| Pax6 | RPCs, ganglion, amacrine, horizontal and müller glia | 1:5 | Mouse monoclonal | Developmental Studies Hybridoma Bank | N |
| Phospho-histone H3 | Mitotic cells (M phase) | 1:200 | Rabbit polyclonal | Upstate Biotechnology 06-570 | N |
| Protein kinase C α | Mature rod bipolar cells | 1:2000 | Mouse monoclonal | Sigma P5704 | N |
| Prox1 | Horizontal, subset of amacrine cells | 1:2000 | Rabbit polyclonal | Covance Research Product PRB-238C | N |
| Pou4f2 (Brn3b) | Transition and mature ganglion cells | 1:50 | Goat polyclonal | Santa Cruz SC-6062 | N |
| Pvalb (Parvalbumin) | Amacrine subtypes | 1:1000 | Mouse monoclonal | Sigma P3088 | N |
| Rb | Ganglion, Müller, Horizontal, amacrein cells (incl. SACs) | 1:100 | Mouse monoclonal | Pharmingen 554136 | Deleted in DKO |
| Rcvrn (Recoverin) | Rods and cone bipolars | 1:100 | Rabbit polyclonal | Chemicon AB5585 | N |
| Rho (RetP1, Rhodopsin) | Rod outer segments | 1:1000 | Mouse Monoclonal | Sigma R5403 | N |
| Rlbp1 (CRALBP) | Late stage precursor and mature Müller | 1:1000 | Rabbit polyclonal | J Saari, U Washington Seattle. | N |
| Sag (Rod arrestin) | Mature rods and cones | 1:50 | Mouse Monoclonal | PA Hargrave, U Florida | N |
| Slc1a3 (GLAST) | Mature Müller | 1:200 | Rabbit polyclonal | Abcam ab416 | N |
| Slc6a9 (GlyT1) | Amacrine subtypes | 1:500 | Goat polyclonal | Chemicon AB1770 | N |
| Slc18a3 (VAChT) | SACs | 1:200 | Goat polyclonal | Promega G448A | A |
| Snap25S | synapses | 1:500 | Mouse monoclonal | S Sugita , U Toronto | N |
| Stx (Syntaxin ) | Amacrine cell processes. | 1:2000 | Mouse monoclonal | Sigma S0664 | N |
| Sv2c | Amacrine subtypes | 1:100 | Rabbit polyclonal | R Janz, UT-Houston Medical School | A |
| Tacr3 (NK3R) | Subset of bipolar cells | 1:500 | Rabbit polyclonal | Abcam AB7 | N |
| Th (tyrosine hydroxylase 2) | Amacrine subtypes | 1:100 | Rabbit polyclonal | Chemicon AB152 | N |
| Vsx1 | Mature cone bipolar cells | 1:50 | Rabbit polyclonal | R. McInnes and RL Chow, U Toronto | N |

*: N: normal staining pattern. **A**: abnormal staining pattern.
