# Supplementary material for: Rb-Mediated Neuronal Differentiation through Cell-Cycle–Independent Regulation of E2f3a
Source: PLoS Biol. 2007 Jul 3;5(7):e179. doi: 10.1371/journal.pbio.0050179 (PMC1914394; doi:10.1371/journal.pbio.0050179)
Supplement: Table S2 — (49 KB DOC) [file pbio.0050179.st002.doc]

**Table S2. Real-time reverse transcript PCR primers**

| Genes | Forward primer (5’ to 3’) | Reverse primer (5’ to 3’) |
| --- | --- | --- |
| *Cdkn2d(p19^Cdkn2d^)* | ATGTCCAAGATGCCTCCGGTA | CATGCTCCACCAGAACCTTCA |
| *Cdkn1a(p21^Cip1^)* | GTGGCCTTGTCGCTGTCTT | GCGCTTGGAGTGATAGAAATCTG |
| *Cdkn1b (p27^Kip1^)* | TCTCTTCGGCCCGGTCAAT | GGGGCTTATGATTCTGAAAGTCG |
| *Cdkn1c( p57^Kip2^)* | CGAGGAGCAGGACGAGAATC | GAAGAAGTCGTTCGCATTGGC |
| *Cdk2* | TCCTCTGAGAGCAGTGATGCA | TTCCCCCAATGACCTAACCAG |
| *Cdk4* | CCAATGTTGTACGGCTGATGG | TGTCCAGGTATGTCCTCAGGT |
| *Ccna2(Cyclin A2)* | GACAGAGCTGGCCTGATGCAT | TGGCGCTTTGAGGTAGGTCT |
| *Ccne1(Cyclin E1)* | TGGCCTATGCAACGACACG | AACATCCAGGACGCACAGGT |
| *E2f1* | CTGCAGCAACTGCAGGAGAG | CTCCGAAAGCAGTTGCAGC |
| *E2f2* | ACGGCGCAACCTACAAAGAG | GTCTGCGTGTAAAGCGAAGT |
| *E2f3a* | GCCTCTACACCACGCCACAAG | CCTTCCAGCACGTTGGTGAT |
| *E2f3b* | CGGAAATGCCCTTACAGC | CCTTCCAGCACGTTGGTGAT |
| *E2f4* | CGCTGACAAGCTGATTGAGCT | AGTGACATTCCGGATGCTCTG |
| *E2f5* | AATGATTACCTGTCCAGCAGCTG | GCTGTAACTGCCCTCTCAGGAG |
| *E2f6* | GCACCAAAGGACCCATTGAT | GCATTGTGGAT GGCTGCTT |
| *E2f7* | GTGCCTTGTGGCTGCTCCT | GCACAGAGTGAACGGACCG |
| *Dhfr* | AGGGAGGGGTAGTTGGAAGA | GGGTGGCTCAGCAGTATAGC |
| *Casp3(Caspase3)* | CTGTACGCGCACAAGCTAGA | CTTTGCGTGGAAAGTGGAGT |
| *Apaf1* | GATGCATTCAAATTGGTTGG | ACCACCATTACCTTGGGACA |
| *Myc(C-myc)* | CAGATCAGCAACAACCGCAA | GACGTTGTGTGTCCGCCTCT |
| *Hprt* | TGTGGCCATCTGCCTAGTAA | GGACGCAGCAACTGACATT |
